# Fine-Tuning-Free Reference-Guided Diffusion for Structure-Preserving Elongated Fabric Defect Generation

**DOI:** 10.3390/s26196351

**Published:** 2026-10-08

**Authors:** Qiang Wu, Hedong Yang, Wensheng Ding, Zhong Xiang, Jinling Yao

**Affiliations:** 1 School of Mechanical Engineering, Zhejiang Sci-Tech University, Hangzhou 310018, China; qiang.wu@zstu.edu.cn (Q.W.); 2024220503187@mails.zstu.edu.cn (H.Y.); xz@zstu.edu.cn (Z.X.); 2 Zhejiang Kangli Automation Technology Co., Ltd., Shaoxing 312000, China; 3 China Textile Machinery Association, Beijing 100028, China; dingwensheng@ctma.net

**Keywords:** industrial anomaly synthesis, elongated fabric defects, fine-tuning-free model, reference-guided generation, latent anchoring

## Abstract

In industrial anomaly detection, anomaly generation helps reduce reliance on scarce real defect samples. However, for elongated fabric defects where morphology is highly coupled with background texture, maintaining the realism of the defects remains a challenge. To address this issue, we propose the Shape-Aware Implicitly Anchored Industrial Defect Generation (SLAID) framework, a reference-image-guided synthesis framework for elongated fabric defects that is fine-tuning-free. Within this framework, we introduce Shape-Sensitive Sliding-Window Reference Modeling (SSRM) to preserve the geometric structure of long-span defects. Additionally, we incorporate Adaptive High-Frequency Guidance (AHFG) to enhance fine edges and textural features. Finally, we propose Target-Domain Latent Anchoring (TLA) to constrain target–background consistency in non-edited regions. SLAID generates structurally continuous defects while preserving the target fabric background, without additional fine-tuning. In a data augmentation experiment on a dataset comprising five categories of elongated textile defects, SLAID improved the mAP@0.5 of YOLOv8 from 0.616 to 0.817. Furthermore, SLAID achieved a mean MMD^2^ of 0.0435 for defect-distribution consistency, a mean LPIPS of 0.0739 for defect-level perceptual similarity, and a full-image SSIM of 0.9754 for target–background preservation. Cross-dataset evaluation on the MHPSD steel-surface defect dataset provides preliminary evidence of the applicability of the proposed inference-stage generation strategy to the evaluated steel-surface defect categories.

## 1. Introduction

Fabric defect detection is a critical component of textile quality control [1,2,3]. However, in actual production, the number of defect samples is far fewer than that of normal samples. This imbalance limits the training of supervised detectors [4] and weakens their generalization capabilities across different fabric textures, production environments, and defect morphologies. To mitigate the reliance on scarce defect samples, high-quality defect synthesis techniques have emerged as a solution to enhance the robustness of detectors in data-scarce scenarios [5,6].

Existing anomaly synthesis methods can generally be divided into rule-based data augmentation, image-editing-based defect injection, and generative model-based synthesis [7,8,9]. Early studies commonly relied on predefined operations, including shape perturbation, texture replacement, copy-paste manipulation, and noise injection [10]. While these approaches are relatively easy to control, the fabric defects they generate often show limited realism in morphology, directional distribution, and background blending.

Recently, defect synthesis has been further advanced by Generative Adversarial Networks (GANs) and diffusion models, leading to notable improvements in both realism and controllability. For instance, CutPaste [11], DRAEM [12], and GLASS [13] improve defect simulation through rule-based perturbation, reconstruction-based discrimination, and expanded defect coverage, respectively. In addition, Mask-Guided Generation [14], Defect-Gen [15], TF-IDG [16], and Structured-GDM [17] further improve the realism and diversity of synthetic defects by incorporating mask constraints, diffusion-based generation, reference-based defect modeling, or structural control.

Nevertheless, fabric defects have domain-specific properties that are different from those of general industrial anomalies. Fabric surfaces usually present repetitive warp-weft textures, and defects such as broken yarns, scratches, streaks, and yarn irregularities are closely related to the underlying texture direction and local illumination changes [18]. In particular, elongated defects may cover a wide spatial range but account for only a small portion of image pixels. Their visual saliency mainly depends on directional continuity, weak boundary responses, and subtle texture deviations from the surrounding background. However, most existing synthesis approaches are primarily designed for general industrial anomalies or localized defects with distinct boundaries. Therefore, the specific requirements of elongated fabric defects, including long-range continuity, subtle high-frequency boundary characteristics, and fine background blending, remain insufficiently investigated.

Reference-guided controllable generation offers an effective solution to this problem. This approach enables the transfer of defect appearances from reference images to specified regions of normal target images without the need to retrain the model for each defect category. The proposed SLAID (Shape-Aware Implicitly Anchored Industrial Defect Generation) method is based on the parameter-frozen AnyDoor framework [19], which provides a fine-tuning-free (parameter-frozen) backbone network for reference-guided image editing. We utilize DINOv2 [20] to extract robust reference features from defect samples and employ spatial conditional control based on ControlNet [21] to provide additional guidance for preserving shape and local details during the diffusion denoising process.

However, directly applying AnyDoor-style object customization to elongated fabric defects remains challenging. These defects usually have high aspect ratios, sparse pixel distributions, and strong directional continuity. When reference regions are normalized to a fixed crop size, elongated structures may be compressed, causing the loss of long-range contextual cues. In addition, the periodic warp-weft textures of fabric surfaces can obscure subtle defect responses, making weak boundaries and fine texture deviations difficult to preserve. During DDIM sampling [22], reference conditions may also affect regions that should remain unchanged, resulting in texture drift, color mismatch, or boundary artifacts.

To address these issues, SLAID introduces three inference-stage components: Shape-Sensitive Sliding-Window Reference Modeling (SSRM) for preserving long-range defect morphology, Adaptive High-Frequency Guidance (AHFG) for enhancing weak boundaries and fine texture cues, and Target-Domain Latent Anchoring (TLA) for stabilizing unedited background regions during sampling. These components jointly improve structural continuity, detail fidelity, and background compatibility without requiring additional fine-tuning of the generation backbone. The main contributions of this work are summarized as follows:

(1) SLAID is presented as a fine-tuning-free reference-guided diffusion framework for synthesizing high-aspect-ratio, low-contrast, and long-span fabric defects.
(2) We design a cooperative sampling strategy that coordinates morphology, detail, and background constraints along the DDIM denoising process. SSRM preserves long-range reference morphology, AHFG converts weak high-frequency defect responses into spatial guidance, and TLA anchors non-edited target latents to suppress texture drift.
(3) SLAID is evaluated on five elongated defect categories from the ZD001 fabric dataset, with additional cross-dataset evaluation on the MHPSD steel-surface defect dataset. The experiments assess generation quality relative to representative synthesis and reference-guided baselines, downstream detection improvements on ZD001, and applicability to the evaluated MHPSD defect categories.

## 2. Related Work

### 2.1. Industrial Anomaly Sample Generation

The generation of industrial anomaly samples aims to address issues such as the scarcity of real anomaly samples, high collection costs, and difficulties in annotation [23]. Recent methods for synthesizing industrial anomalies can be categorized into the following four representative paradigms: manually designed methods, methods based on distribution assumptions, methods based on generative models, and methods based on vision-language models [24]. Although significant progress has been made in this field, the number of available defect samples remains limited in many practical detection scenarios. This limitation makes it difficult to fully capture the diversity of real industrial anomalies in terms of morphology, appearance, and spatial distribution, particularly in scenarios involving small-batch production and high-quality annotation requirements [9].

Early generative methods largely relied on GAN-based (Generative Adversarial Network) image transformation or defect injection strategies to learn defect appearance features and the distribution of their surrounding backgrounds. Niu et al. [25] improved defect recognition performance by synthesizing additional defect samples, while Defect-GAN decomposed the generation process into two stages, namely defect insertion and image restoration, to better preserve background consistency [26]. While these methods can mitigate class imbalance in few-shot detection to some extent, they typically require training on specific datasets. Furthermore, their generation quality is largely constrained by the diversity and representativeness of existing defect samples.

This limitation is particularly pronounced in the case of elongated fabric defects. Such defects typically occupy only a small number of pixels in an image but may extend over long spatial distances and often distribute along the warp or weft directions of periodic textile textures. With limited training samples, generative models may only capture local, short-segment features, making it difficult to reconstruct the complete topological structure of the defect. Consequently, synthetic images are prone to issues such as broken boundaries, local texture repetition, and unnatural transitions between defect and background regions.

In recent years, advancements in few-shot learning and diffusion models have further enhanced the controllability of industrial anomaly synthesis. Duan et al. [27] generated few-shot defect images through defect-aware feature operations. AnomalyXFusion introduced multimodal conditional control to enhance appearance diversity [28]; while AnomalyDiffusion utilizes latent diffusion priors and spatial anomaly embeddings to synthesize few-shot anomaly samples aligned with masks [29]. CAGEN combines text prompts with mask conditions to achieve more precise control over the location and type of anomalies [30]. RealNet further introduces an intensity-controllable diffusion-based anomaly synthesis strategy to generate realistic anomaly samples for detection tasks [31]. Although these methods improve the realism and controllability of anomaly generation, most still focus on local image patches, texture perturbations, or general surface defects. For elongated fabric defects, key characteristics such as the dominant direction, spatial span, skeletal continuity, faint high-frequency boundaries, and compatibility with periodic fabric backgrounds remain insufficiently addressed. To address this gap, we propose a dedicated synthesis strategy for elongated fabric defects.

### 2.2. Reference-Based Fine-Tuning-Free Diffusion Generation

Diffusion models have become a crucial technological foundation for controllable image generation and editing. DDPM established a generative modeling framework centered on incremental denoising [32]; building on this, DDIM improved sampling efficiency and provided better trajectory controllability for the denoising process [22]; latent diffusion models, meanwhile, transfer the diffusion process to a compressed latent space, thereby reducing computational overhead [33]. In the fields of industrial anomaly detection and defect synthesis, diffusion models are also increasingly being used for tasks such as anomaly sample generation, data augmentation, and anomaly region reconstruction [34].

Local image editing and image restoration methods typically rely on conditions such as text prompts, masks, or degraded images to constrain the editing region and generated content. Studies such as Blended Diffusion [35], DiffusionCLIP [36], RePaint [37], and Palette [38] demonstrate that diffusion priors can support structure-preserving local editing under explicit spatial or semantic constraints. However, these methods are primarily designed for natural image editing, semantic attribute manipulation, and general-purpose image restoration, and thus differ from the requirements of industrial defect synthesis regarding defect morphology, material consistency, and background blending.

Reference- or subject-guided diffusion methods further leverage external examples to constrain the visual appearance of the generated results. DreamBooth customizes text-to-image diffusion models for specific subjects using a small number of reference images [39]. This method effectively preserves the features of the target subject but typically requires additional model fine-tuning. In contrast, fine-tuning-free reference-guided editing methods such as AnyDoor [19] and TF-IDG [16] reduce model adaptation costs by transferring appearance information or defect-related features from reference images during the inference stage. This feature is of practical significance for industrial defect synthesis, as each defect category often provides only a limited number of available samples.

However, general reference-guided editing methods remain difficult to apply directly to elongated fabric defects. Most existing methods focus on semantic object replacement, compact region editing, or natural image restoration, typically assuming that the region to be edited possesses a relatively complete and concentrated appearance structure. Elongated fabric defects do not satisfy this assumption. Such defects often exhibit characteristics such as narrowness, low contrast, and long-range extension. When only a single normalized reference crop is used, the main-axis structure of elongated defects may be compressed, high-frequency boundary information may be weakened, and long-range contextual cues may be lost. Furthermore, during the diffusion sampling process, reference conditioning may inadvertently affect the fabric background, which is expected to remain unchanged, resulting in texture drift, color mismatch, or boundary artifacts. Therefore, shape- aware modeling, high-frequency structural guidance, and latent spatial constraints on the target region are necessary to better support the fine-tuning-free generation of elongated fabric defects.

## 3. Methods

### 3.1. Overview of Shape-Aware Implicitly Anchored Industrial Defect Generation Framework

**Figure 1:**
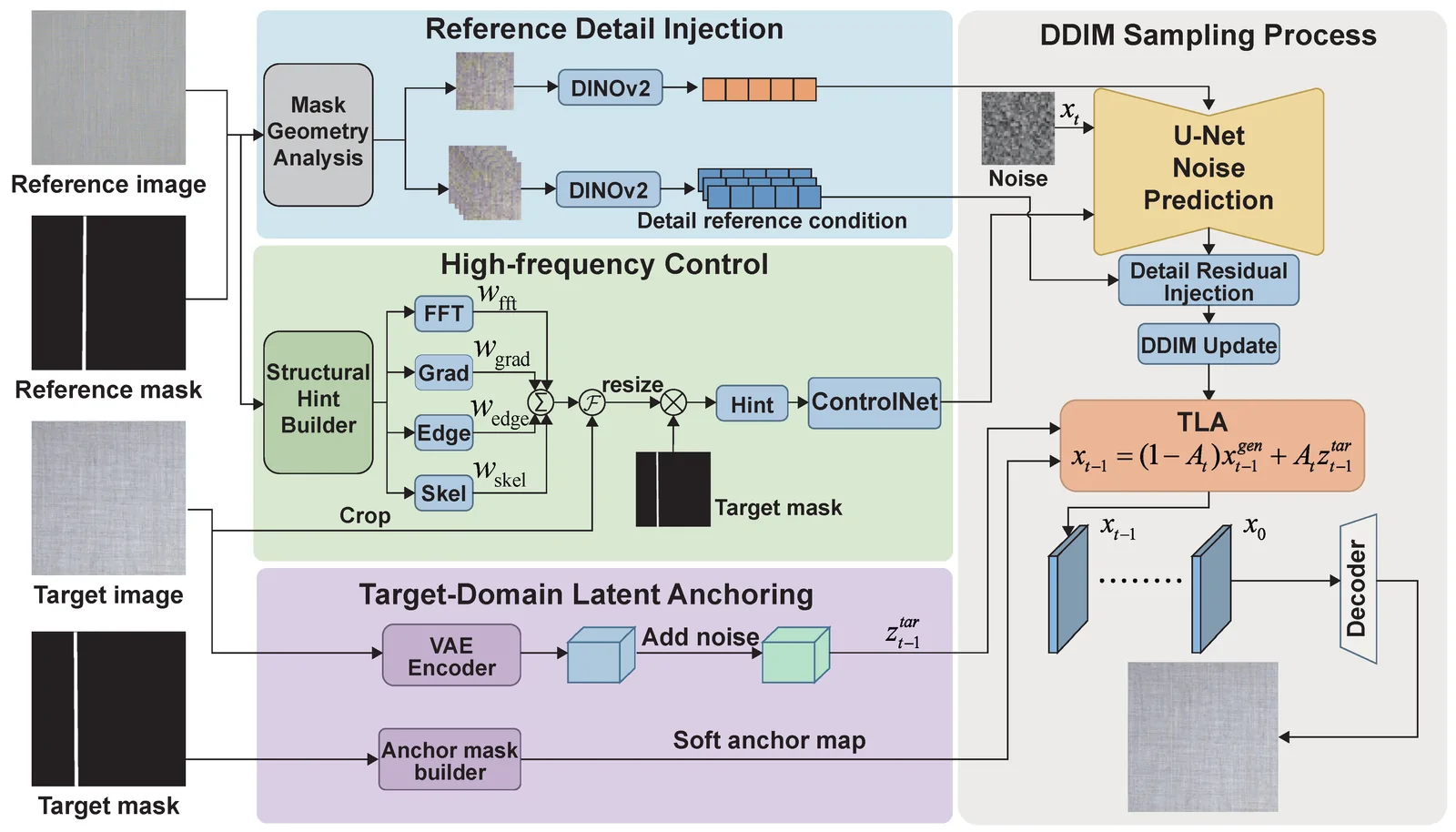
Overview of the proposed SLAID framework. Given a reference defect image with its mask and a normal target image with a target mask, SLAID generates elongated fabric defects through three fine-tuning-free components: shape-sensitive sliding-window reference modeling, adaptive high-frequency guidance, and defect-protected target-domain latent anchoring during DDIM sampling.

The generation of elongated fabric defects requires the simultaneous preservation of long-range morphology, fine defect details, and consistency with the target fabric background. To this end, SLAID is designed as a fine-tuning-free, reference-guided diffusion framework for elongated fabric defect synthesis. In this paper, “fine-tuning-free” means that no additional optimization is performed on the target dataset. SLAID uses pretrained AnyDoor [19], DINOv2 [20], ControlNet [21], and VAE [33] models with frozen parameters, and all proposed modules operate only during inference. As shown in Figure 1, SLAID takes a reference defect image $I_{r}$ with its mask $M_{r}$, and a normal target fabric image $I_{t}$ with a target mask $M_{t}$ as inputs. It transfers the defect structure and appearance from the reference image to the specified target region, producing a synthetic defective image $\hat{I}$.

The process includes three components. Shape-Sensitive Sliding-Window Reference Modeling (SSRM) builds a reference representation according to the geometry of the reference defect, which helps preserve the long-span morphology of slender structures. Adaptive High-Frequency Guidance (AHFG) extracts structural and high-frequency cues to strengthen weak boundaries and fine defect textures. Target-Domain Latent Anchoring (TLA) constrains the DDIM sampling trajectories within the unedited target region, thereby reducing background drift and boundary artifacts in the latent space.

Algorithm 1 summarizes the inference workflow of SLAID. The three components are introduced only at the inference stage, while the original AnyDoor model is kept frozen during the whole process. Therefore, SLAID can generate structurally continuous and background-consistent elongated fabric defects without additional model training or fine-tuning.
**Algorithm 1** Fine-tuning-free inference process of SLAID**Require:** 
Frozen AnyDoor model $D_{\theta }$, DDIM sampler, reference image $I_{r}$, reference mask $M_{r}$, target image $I_{t}$, target mask $M_{t}$**Ensure:** 
Synthetic elongated fabric defect image $\hat{I}$  1:Estimate defect geometry from $M_{r}$.  2:Build shape-aware reference representations R using SSRM.  3:Extract adaptive high-frequency guidance *H* using AHFG.  4:Compose target control hint $C_{t}$ from $I_{t}$, $M_{t}$, and *H*.  5:Encode reference representations into conditions $c_{r}$.  6:Encode target crop into latent $z_{t}^{0}$ and construct anchor mask $M_{\mathrm{anc}}$.  7:Initialize DDIM latent $x_{T}$ with target-domain anchoring.  8:**for** τ=T to 1 **do**  9:      Predict noise $\epsilon_{\theta }\left(x_{\tau },C_{t},c_{r}\right)$ with the frozen AnyDoor model.10:      Apply shape-aware detail guidance from R.11:      Perform one DDIM denoising step to obtain $x_{\tau -1}$.12:      Anchor non-edited regions in latent space using TLA.13:**end for**14:Decode $x_{0}$ and blend the generated patch back into $I_{t}$.15:**return** $\hat{I}$.

### 3.2. Shape-Sensitive Sliding-Window Reference Modeling (SSRM)

Slender fabric defects usually show large aspect ratios, sparse pixel distributions, and clear directional continuity. Using a single normalized reference patch to represent these defects makes it difficult to retain fine texture cues and long-range topology simultaneously, especially when the defect covers a large part of the image. To reduce this limitation, SSRM builds shape-aware reference representations based on the geometric properties of the reference defect.

Given a reference image $I_{r}$ and its defect mask $M_{r}$, SSRM first estimates the main defect direction, and then computes the major axis length $L_{\mathrm{maj}}$, the minor axis length $L_{\min}$, and the image scale along the major direction $L_{\mathrm{img}}$. The spatial span ratio and aspect ratio are defined as:

(1) $$\rho =\frac{L_{\mathrm{maj}}}{L_{\mathrm{img}}},\;\eta =\frac{L_{\mathrm{maj}}}{L_{\min}+\epsilon }.$$

When both ρ and η are large, the defect is treated as a long-span, slender structure, and SSRM generates multiple overlapping windows along its major axis. Otherwise, SSRM employs a locally enhanced reference representation to improve the detectability of narrow defects at a fixed input scale.

In the sliding window branch, candidate windows are scored based on their internal defect responses. Concurrently, a central dispersion constraint is introduced to ensure that the selected windows cover different segments of the slender structure, avoiding concentration in a single local region. Let $s_{n}$ denote the response score of the *n*th selected window; its reference weight is then calculated as:

(2) $$\alpha_{n}=\frac{s_{n}}{\sum_{j}s_{j}+\epsilon }.$$

Before being fed into the reference encoder, each selected window undergoes context padding, scale normalization, and fixed-size scaling. Concurrently, SSRM records the relative positions of each window along the main direction and constructs an object assignment map for subsequent spatial detail allocation.

To minimize the loss of window-specific details during the reference encoding process, SSRM further introduces a window-level detail adjustment mechanism into the U-Net denoising process. First, a baseline noise prediction is obtained using the global reference condition $c_{g}$. Subsequently, a local noise prediction is generated for each sliding window condition $c_{n}$, and the difference between this and the global prediction is treated as the detail residual:

(3) $$\Delta \varepsilon_{n}=\varepsilon_{\theta }\left(x_{t},t,c_{n}\right)-\varepsilon_{\theta }\left(x_{t},t,c_{g}\right),$$

where $c_{g}$ and $c_{n}$ denote the global reference condition and the *n*th sliding window reference condition, respectively. The detail residuals are incorporated into the denoising process based on the spatial distribution map $A_{n}$, the reliability weight $r_{n}$, and the time-step-dependent coefficient $\lambda_{t}$:

(4) $${\tilde{\varepsilon }}_{t}=\varepsilon_{g}+\lambda_{t}\sum_{n}r_{n}A_{n}\Delta \varepsilon_{n}.$$

With this design, SSRM can strengthen the local texture response of narrow defects while preserving the structural continuity of large-span defects. The obtained reference conditions contain both global topological information and local detail cues, thereby providing more stable structural guidance for elongated fabric defect generation.

### 3.3. Adaptive High-Frequency Guidance (AHFG)

SSRM offers shape-aware reference representations, whereas slender fabric defects still rely heavily on subtle high-frequency cues. Contours of broken yarns, stripe edges, and weak texture variations are usually sparse and have low contrast. When generation depends only on reference appearance conditions, such cues may become blurred or weakened during the denoising process. To address this issue, AHFG extracts frequency and structural information from representative reference windows and transforms it into spatial control signals.

Given a selected reference patch and the corresponding defect mask, AHFG first builds a defect-aware support region. The mask is slightly eroded to reduce unstable boundary noise. It is then dilated with a direction-sensitive rectangular structural element aligned with the main defect direction. The obtained strip-like support region *S* covers the defect together with its neighboring areas, while reducing the influence of irrelevant background textures.

Within *S*, four complementary responses are calculated. The first one is the frequency-domain periodic residual, which emphasizes local disturbances in periodic fabric textures. Let $I_{g}$ represent the grayscale cropped reference image, and let F(·) represent the Fourier transform. After the dominant periodic peaks are suppressed by a notch filter *A*, the residual response is defined as:

(5) $$R_{\mathrm{fft}}=N_{S}\left(\left|I_{g}-F^{-1}\left(A\circ F(I_{g})\right)\right|\right),$$

where ∘ denotes element-wise multiplication, and $N_{S}\left(\cdot \right)$ denotes normalization within the support domain. The second response measures the local gradient using the Sobel operator:

(6) $$R_{\mathrm{grad}}=N_{S}\left(|\partial_{x}I_{g}\left|\;+\;\right|\partial_{y}I_{g}|\right).$$

The third response $R_{\mathrm{edge}}$ comes from the morphological gradient of the defect mask and highlights the defect contours. The fourth response $R_{\mathrm{ctr}}$ is extracted via morphological skeletonization to preserve the connectivity of fine-line structures.

These responses are fused into a composite high-frequency map:

(7) $$R=\omega_{f}R_{\mathrm{fft}}+\omega_{g}R_{\mathrm{grad}}+\omega_{e}R_{\mathrm{edge}}+\omega_{c}R_{\mathrm{ctr}},$$

where $\omega_{f}$, $\omega_{g}$, $\omega_{e}$, and $\omega_{c}$ are fusion coefficients. AHFG does not employ a fixed global threshold. Instead, the activation threshold $\tau_{h}$ is determined based on the distribution of responses within the defect region. The binary activation map is obtained via the following equation:

(8) $$B=\max\left(1\left(R\geq \tau_{h}\right),R_{\mathrm{ctr}}\right),$$

where the skeleton response is retained to reduce discontinuities in thin defects.

The sparse activation map is then fused with and smoothed against the continuous response map to generate the high-frequency intensity map:

(9) $$V=\mathrm{Blur}\left(\lambda_{b}B+\lambda_{r}R\right),\;\lambda_{b}+\lambda_{r}=1.$$

To preserve the color appearance of the reference defect, *V* is written to the luminance channel of the HSV color space, while the original hue and saturation remain unchanged. The resulting high-frequency guide map $H_{\mathrm{hf}}$ is used as part of the spatial control conditions for defect generation.

Unlike traditional edge maps or fixed high-pass filters, AHFG combines periodic texture residuals, local gradients, defect boundaries, and centerline structures. Consequently, this guidance information encodes both the locations where defects should appear and how their subtle high-frequency details should be represented. This helps improve the sharpness, continuity, and detectability of the generated elongated fabric defects.

### 3.4. Target-Domain Latent Anchoring (TLA)

SSRM and AHFG provide structural and high-frequency guidance, but diffusion sampling may still disturb fabric regions outside the edit area. This problem is especially evident for narrow defects, where the editable region is small and the surrounding periodic texture should remain unchanged. When reference conditions are applied directly, non-edited regions may suffer from texture drift, color mismatch, or boundary artifacts. To address this issue, TLA constrains the non-edited regions along the DDIM denoising trajectory in the target latent space.

Given the target crop $I_{t}^{c}$, it is first encoded into the latent space by the frozen first-stage encoder:

(10) $$z_{0}^{t}=E\left(I_{t}^{c}\right),$$

where E(·) denotes the VAE encoder. A soft edit mask is then constructed from the target mask $M_{t}$. The target mask is cropped, dilated with a core kernel and a larger ring kernel, smoothed by Gaussian filtering, padded to a square shape, and resized to the latent resolution. The resulting edit weight is denoted as $M_{\mathrm{edit}}$, and the initial anchor weight is defined as:

(11) $$M_{\mathrm{anc}}^{0}=1-M_{\mathrm{edit}}.$$

Thus, $M_{\mathrm{anc}}^{0}$ has high responses in non-edited regions and low responses in the defect core.

TLA also prevents defect-related regions from being over-anchored, since these regions need enough freedom for reference-guided generation. Let $M_{\mathrm{reg}}$ and $M_{\det}$ denote the target region weight and detail weight produced by the previous modules. The protection map is constructed as:

(12) $$M_{\mathrm{pro}}=\max\left(M_{\mathrm{reg}},M_{\det}\right),$$

and the final anchor mask is obtained by:

(13) $$M_{\mathrm{anc}}=\mathrm{clip}\left(M_{\mathrm{anc}}^{0}⊙\left(1-\beta M_{\mathrm{pro}}\right),0,1\right),$$

where β controls the protection strength. As a result, non-edited background regions remain strongly anchored, while defect and detail regions keep sufficient generation freedom.

Before DDIM sampling, TLA initializes the latent variable from the target-domain noisy trajectory. Given Gaussian noise ϵ∼N(0,I), the noisy target latent at timestep *T* is computed as:

(14) $$z_{T}^{t}=\sqrt{{\bar{\alpha }}_{T}}z_{0}^{t}+\sqrt{1-{\bar{\alpha }}_{T}}\epsilon .$$

The initial DDIM latent is then defined as:

(15) $$x_{T}=\left(1-M_{\mathrm{anc}}\right)⊙\epsilon +M_{\mathrm{anc}}⊙z_{T}^{t}.$$

In this initialization, the editable defect region starts from random noise, whereas the non-edited background starts from the noisy target latent distribution.

During each DDIM denoising step, after the intermediate latent $x_{\tau -1}$ is obtained, TLA computes the corresponding target-domain noisy latent:

(16) $$z_{\tau -1}^{t}=\sqrt{{\bar{\alpha }}_{\tau -1}}z_{0}^{t}+\sqrt{1-{\bar{\alpha }}_{\tau -1}}\epsilon .$$

The non-edited regions are then anchored as:

(17) $$x_{\tau -1}\leftarrow \left(1-\gamma_{\tau }M_{\mathrm{anc}}\right)⊙x_{\tau -1}+\gamma_{\tau }M_{\mathrm{anc}}⊙z_{\tau -1}^{t},$$

where $\gamma_{\tau }$ denotes a timestep-dependent anchoring strength. This parameter controls the trade-off between preserving the target background and allowing local defect generation. A larger $\gamma_{\tau }$ enforces stronger consistency with the original target latent in non-edited regions, whereas a smaller value leaves more freedom around the editable defect region.

With TLA, the editable region is mainly controlled by reference-guided generation, while the non-edited fabric background is repeatedly aligned with the target-domain noisy latent trajectory. This process preserves the original fabric texture outside the edit mask and reduces texture drift, color inconsistency, and boundary artifacts. It improves the background compatibility of elongated fabric defect generation.

## 4. Experiments

### 4.1. Datasets

Two publicly available industrial defect datasets, ZD001 [40] and MHPSD [41], were used to evaluate the proposed method. ZD001 served as the primary dataset for method evaluation and ablation studies, while MHPSD was used for additional cross-dataset evaluation of defect generation.

**Figure 2:**
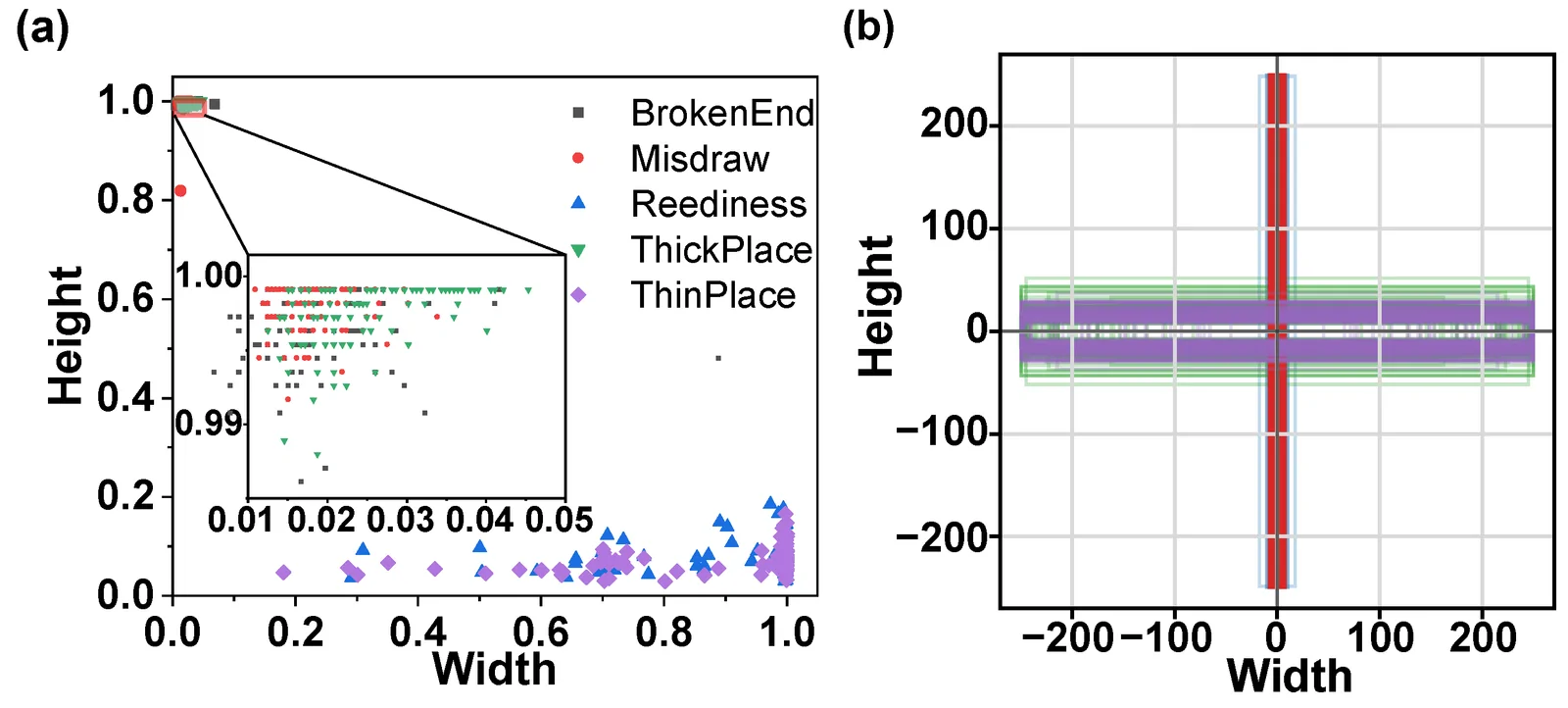
Geometric distribution of selected elongated defect annotations in the ZD001 dataset. (**a**) Scatter plot of normalized bounding-box width and height, where each annotation is normalized by the corresponding image size; the inset magnifies the narrow vertical-defect region. (**b**) Center-aligned overlay of defect bounding boxes in pixel coordinates, with each box translated to a common origin. The two views show that the selected categories contain high-aspect-ratio defects with either near-full-height narrow regions or near-full-width narrow regions.

ZD001 is a publicly available fabric defect dataset collected from quality-inspection stations of multiple textile manufacturers. It contains cotton- and polyester-based fabrics with diverse textile structures and exhibits variations in warp/weft density, yarn count, fabric thickness, production speed, and illumination conditions. Therefore, the images were not acquired under strictly identical conditions, but instead reflect the variability encountered in practical textile inspection. In this study, five predefined categories with representative elongated morphologies were considered, namely BrokenEnd, Misdraw, Reediness, ThickPlace, and ThinPlace. To reduce potential selection bias and ensure balanced category representation, 200 real defect images were randomly sampled within each category and divided into mutually exclusive training, validation, and test subsets containing 100, 50, and 50 images, respectively. The original category labels and detection annotations provided by ZD001 were retained without reassignment or modification. The geometric characteristics of the selected samples are further illustrated in Figure 2.

Although ZD001 includes images collected from multiple textile manufacturers under varying acquisition conditions, explicit machine- and production-batch-level identifiers are not available. The training, validation, and test subsets are mutually exclusive at the image level, but their separation by machine or production batch cannot be verified. Therefore, the current split does not establish performance on unseen machines or production batches.

**Figure 3:**
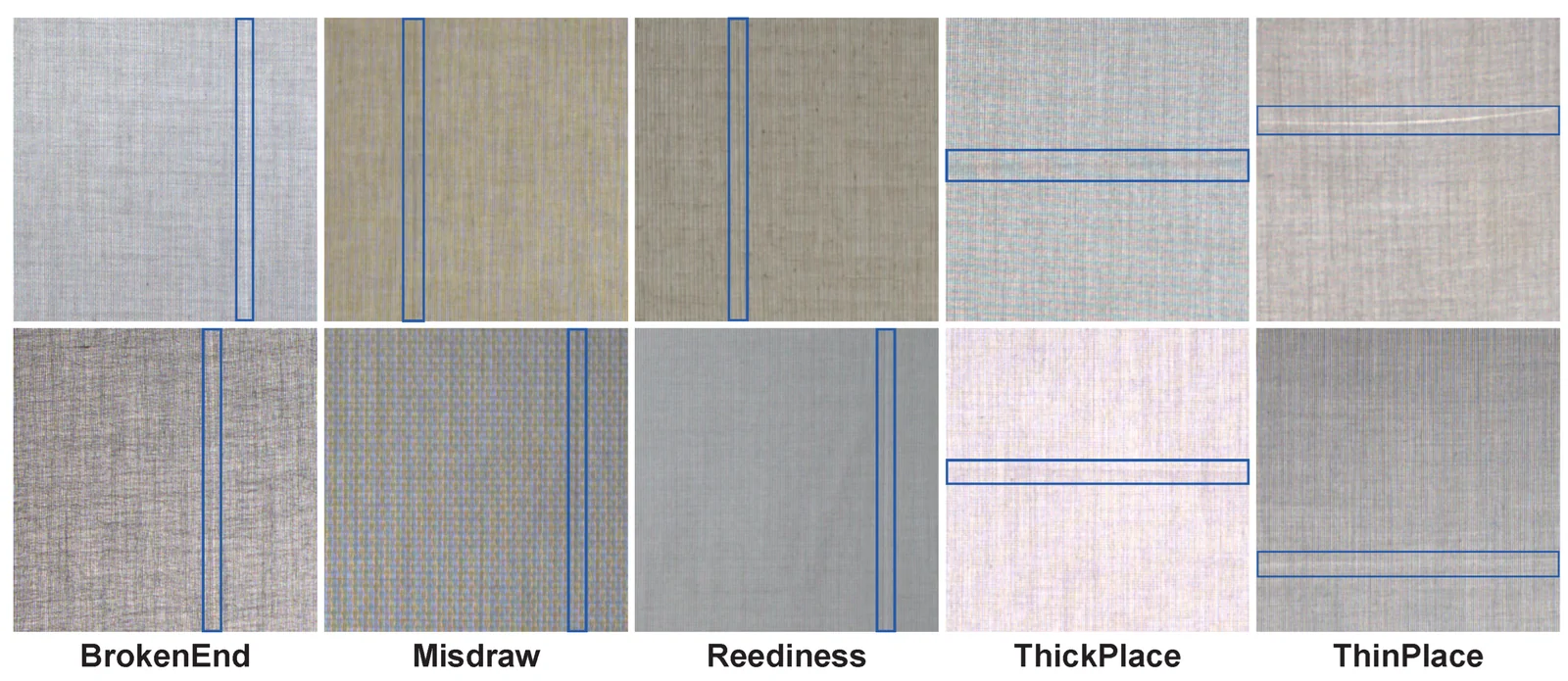
Representative examples of the selected elongated defect categories from the ZD001 dataset. The five categories are BrokenEnd, Misdraw, Reediness, ThickPlace, and ThinPlace, with manually annotated defect regions highlighted by blue boxes.

Since ZD001 provides detection annotations but does not contain pixel-level defect masks, the 500 training images were additionally annotated with pixel-level masks by two annotators with machine-vision experience following a unified annotation protocol. To assess annotation reliability, a stratified subset of 100 images, including 20 images from each category, was independently re-annotated by both annotators, yielding mean Dice and IoU scores of 0.934 and 0.876, respectively. The resulting masks were used as reference and spatial conditions for defect generation, while the detection annotations of the synthetic samples were automatically derived from the corresponding masks. In addition, 300 normal fabric images were selected as target backgrounds. For each defect category, 200 synthetic images were generated and combined with the 100 real training images, resulting in 300 training images per category after augmentation. The validation and test sets remained unchanged and contained only real images. The resulting sample configuration is summarized in Table 1, while representative examples of the five selected defect categories are shown in Figure 3.

**Figure 4:**
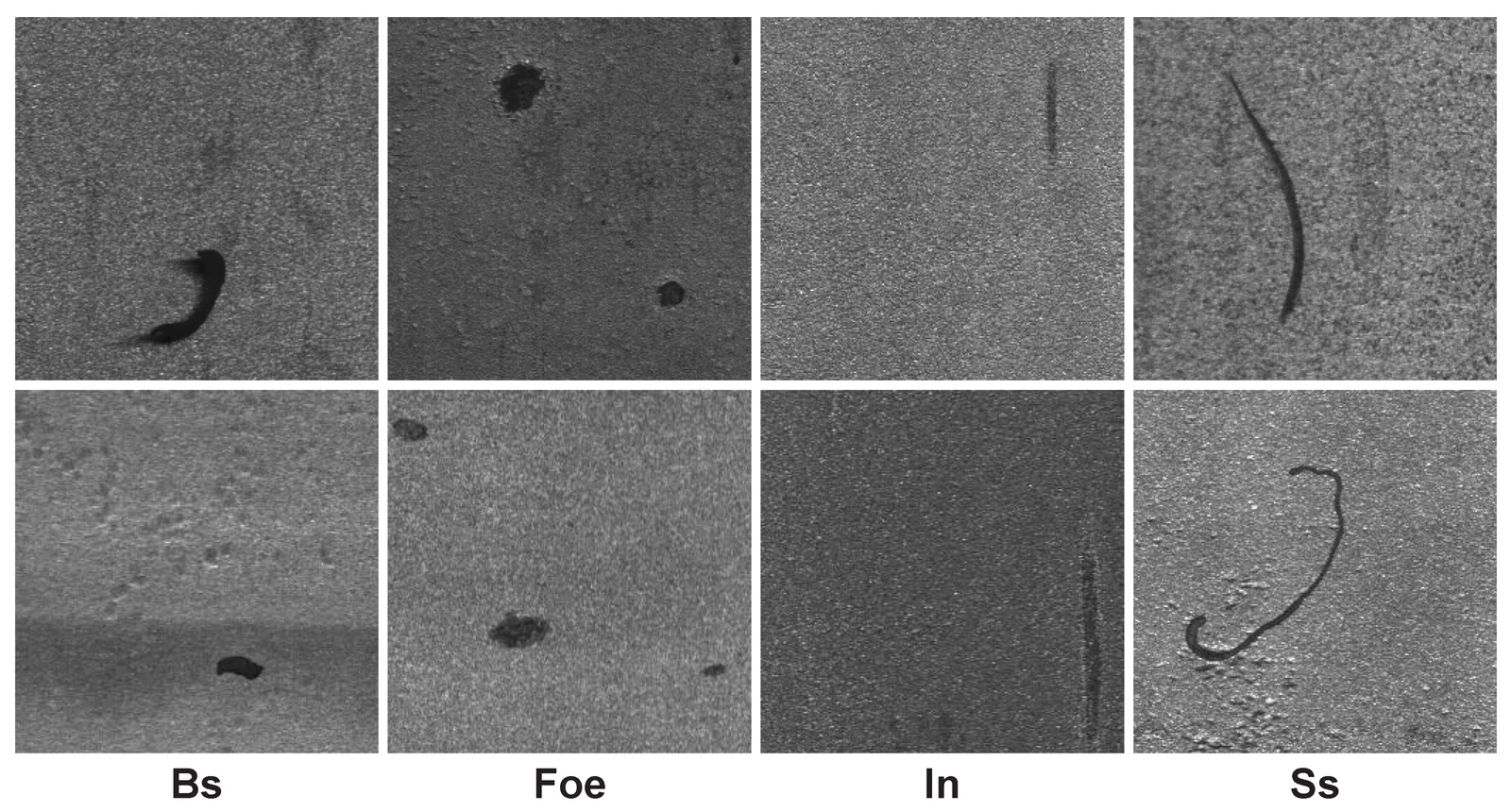
Representative examples of the four surface-defect categories from the MHPSD dataset, including Bs, Foe, In, and Ss.

MHPSD [41] is a Medium and Heavy Plate Surface Defect Dataset collected from surface-quality inspection equipment on real steel production lines. The originally acquired grayscale images have a resolution of 4096 × 3000 pixels and were cropped around localized defect regions to obtain standardized 256 × 256 samples. The dataset contains four representative steel-surface defect categories, namely Blocky Scale (Bs), Striated Scale (Ss), Foreign Object Embedding (Foe), and Inclusions (In), together with defect-free background images. The samples exhibit substantial intra-class morphological diversity, illumination variation, and background-texture variation, providing an industrial domain substantially different from the textile surfaces in ZD001. Representative samples from the four defect categories are shown in Figure 4. In our cross-dataset experiment, 120 real defect images were available for each category, and 200 synthetic images were generated per category, resulting in 320 samples per category after synthetic expansion. The corresponding sample configuration is summarized in Table 2. MHPSD was not used for model training, fine-tuning, or parameter optimization of SLAID.

### 4.2. Metrics

SLAID was evaluated in terms of generation quality and downstream detection performance. Generation quality was measured by Maximum Mean Discrepancy (MMD) [42], Learned Perceptual Image Patch Similarity (LPIPS) [43], and Structural Similarity Index Measure (SSIM) [44]. MMD measures the feature-space distribution gap between generated and real defect images. LPIPS measures the local perceptual similarity between generated defects and their corresponding real reference defects. SSIM measures how well the generated image preserves the original target background.

For MMD, image-level features were extracted from generated and real defect images using an ImageNet-pretrained ResNet-50 [45]. The final classification layer was removed, and the 2048-dimensional vector after global average pooling was used as the image representation. Let $R={\left\{r_{i}\right\}}_{i=1}^{m}$ and $G={\left\{g_{j}\right\}}_{j=1}^{n}$ denote the feature sets of real and generated defect images, respectively. With a Gaussian radial basis function (RBF) kernel $k\left(x,y\right)=\exp(-\gamma ∥x-y{∥}_{2}^{2})$, MMD^2^ is computed as:

(18) $${\mathrm{MMD}}^{2}\left(R,G\right)=\frac{1}{m^{2}}\sum_{i,j=1}^{m}k\left(r_{i},r_{j}\right)+\frac{1}{n^{2}}\sum_{i,j=1}^{n}k\left(g_{i},g_{j}\right)-\frac{2}{mn}\sum_{i=1}^{m}\sum_{j=1}^{n}k\left(r_{i},g_{j}\right).$$

The kernel bandwidth γ was estimated by the median heuristic for each defect category and kept fixed across all compared generation methods. A lower MMD^2^ indicates a smaller feature-space distribution gap between generated and real defect images.

SSIM was used to evaluate background preservation. Each generated image was paired with its corresponding original background image based on matching filenames. SSIM was computed over the full RGB image by evaluating the three color channels independently with a local window size of 7×7. The image-level SSIM score was then obtained by averaging the channel-wise scores:

(19) $${\mathrm{SSIM}}_{RGB}\left(I_{g},I_{o}\right)=\frac{1}{3}\sum_{c=1}^{3}\mathrm{SSIM}\left(I_{g}^{c},I_{o}^{c}\right),$$

where $I_{g}$ and $I_{o}$ denote the generated image and the original target background image, respectively. A higher SSIM indicates better preservation of the non-edited fabric background.

The LPIPS was used to quantify the local perceptual similarity between the generated defects and their corresponding real reference defects. For each generated image $G_{i}$, the bounding box of the corresponding defect mask was expanded by 10% in width and height to include a small amount of surrounding context. The corresponding local regions were then cropped from the generated image and the real reference image $R_{i}$, and resized to the same spatial resolution. Let ${\tilde{G}}_{i}$ and ${\tilde{R}}_{i}$ denote the resulting local defect-context regions, respectively. These regions were then fed into a pretrained perceptual network to extract deep feature representations. Specifically, given the normalized feature activations ${\hat{\phi }}^{l}\left({\tilde{G}}_{i}\right)$ and ${\hat{\phi }}^{l}\left({\tilde{R}}_{i}\right)$ at the *l*-th layer, the LPIPS distance is defined as:

(20) $$\mathrm{LPIPS}\left({\tilde{G}}_{i},{\tilde{R}}_{i}\right)=\sum_{l}\frac{1}{H_{l}W_{l}}\sum_{h,w}{∥w_{l}⊙\left({\hat{\phi }}^{l}{\left({\tilde{G}}_{i}\right)}_{hw}-{\hat{\phi }}^{l}{\left({\tilde{R}}_{i}\right)}_{hw}\right)∥}_{2}^{2}.$$

where ${\hat{\phi }}^{l}\left(\cdot \right)$ denotes the channel-wise normalized feature activation, $w_{l}$ represents the learned channel-wise weights, and $H_{l}$ and $W_{l}$ denote the spatial dimensions of the feature map at the *l*-th layer. A lower LPIPS value indicates greater perceptual similarity between the generated and reference local defect-context regions. The LPIPS values were averaged over all generated samples within each defect category, and the category-wise mean and overall mean were reported.

For downstream detection, Precision (P), Recall (R), Average Precision (AP), and mean Average Precision (mAP) were adopted:

(21) $$\begin{array}{c} P=\frac{TP}{TP+FP},\;R=\frac{TP}{TP+FN}, \end{array}$$

(22) $$\begin{array}{c} \mathrm{mAP}=\frac{1}{C_{d}}\sum_{c=1}^{C_{d}}{\mathrm{AP}}_{c}, \end{array}$$

where $C_{d}$ is the number of defect categories. Higher P, R, AP, and mAP indicate better downstream detection performance.

### 4.3. Experimental Implementation Details

#### 4.3.1. Implementation Details

The experimental environment used for defect generation is summarized in Table 3. SLAID was implemented on the pretrained AnyDoor framework, and all parameters of the original generation backbone were kept frozen during inference. Images were processed at 512×512 during DDIM sampling, and reference conditions were encoded at 224×224. The DDIM sampler used 50 steps with η=0. The classifier-free guidance scale was set to 5.0, and the ControlNet scale was fixed to 1.0.

For SSRM, the maximum number of sliding windows was set to 3. The span-ratio and aspect-ratio thresholds were set to 0.93 and 12.0, respectively. For AHFG, the number of dominant frequency peaks, notch radius, and retained high-frequency response percentage were set to 8, 3, and 45%, respectively. For TLA, the anchor protection strength and latent anchoring strength were set to 0.92 and 0.42. For each defect category, 100 real defective images were used as references, 300 normal images were used as candidate target backgrounds, and 200 synthetic images were generated for training-data augmentation. No additional training or fine-tuning was performed during generation. Our code is publicly available at https://github.com/Kongbai-11/SLAID (accessed on 30 September 2026).

For downstream defect detection, YOLOv8 was trained using the hyperparameters in Table 4. The same detector configuration was used for both the real-data baseline and the SLAID-augmented setting.

#### 4.3.2. Compared Methods

SLAID was compared with four representative baselines covering rule-based synthesis, diffusion-based anomaly generation, reference-guided editing, and fine-tuning-free industrial defect generation. Crop-Paste [46] transfers cropped defect regions onto normal backgrounds. AnoDiff denotes AnomalyDiffusion [29], which learns anomaly-specific representations from limited defect samples. AnyDoor [19] is a reference-guided zero-shot image-editing framework and is used as the direct backbone baseline. TF-IDG [16] is a fine-tuning-free industrial defect generation method based on diffusion priors. All methods were compared under the same defect categories and target normal fabric images.

For downstream evaluation, SLAID-generated images were added to the YOLOv8 training set [47]. All detection models used the same preprocessing, input resolution, data augmentation, and training schedule, and were evaluated on the same real fabric defect test set.

To improve comparability, all methods followed the same data protocol. For AnoDiff, the official implementation was adapted to ZD001 using 100 original, unaugmented real defect images per category. AnyDoor and TF-IDG used their pretrained models without additional fine-tuning, while SLAID kept its generation backbone frozen. The validation and test sets were not used for model adaptation or synthetic sample generation. For methods requiring normal backgrounds, the same pool of 300 normal images was used, and each method generated 200 synthetic images per category.

Despite the shared data and evaluation protocol, the compared methods differ in their pretraining, adaptation requirements, and conditioning mechanisms. These differences cannot be fully controlled within the current comparison. The results should therefore be interpreted as a comparison of the configured methods under the stated experimental conditions, rather than as evidence of superiority independent of their underlying assumptions.

### 4.4. Geometric Consistency and Visual Diversity of Synthesized Defects

**Figure 5:**
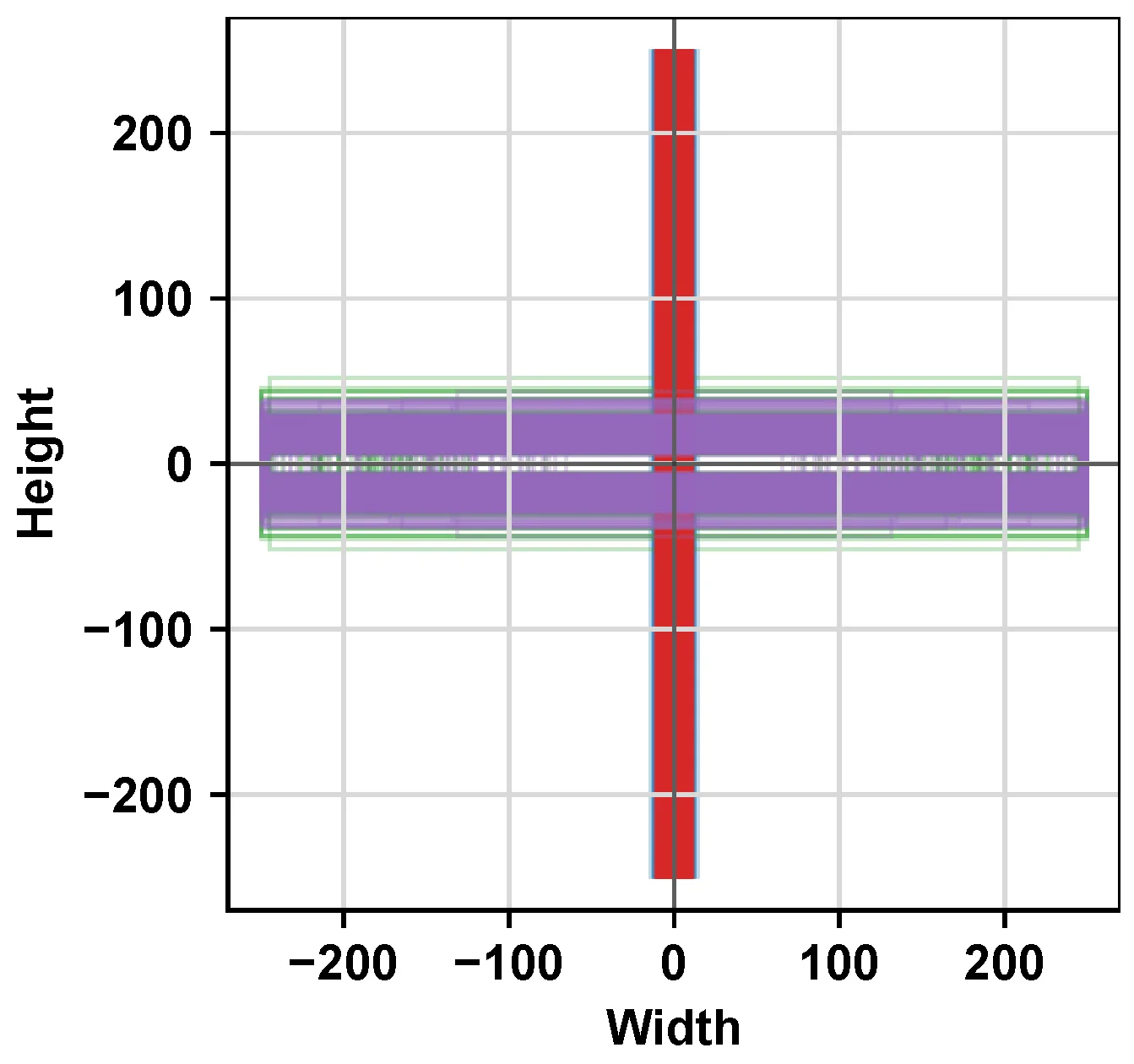
Geometric distribution of the augmented elongated-defect set. Compared with the real-defect geometry in Figure 2, the generated defects show similar elongated bounding-box patterns and spatial extents, covering both vertical and horizontal elongated structures.

We further examined whether the generated defects preserve the geometric characteristics of real elongated fabric defects while increasing the diversity of the training set. Compared with the real-defect distribution shown in Figure 2, the augmented samples in Figure 5 show similar elongated patterns in aspect ratio, defect size, and spatial extent. The generated defects cover both near-vertical and near-horizontal structures, suggesting that the synthetic expansion preserves the geometry of real industrial fabric annotations rather than introducing random perturbations.

**Figure 6:**
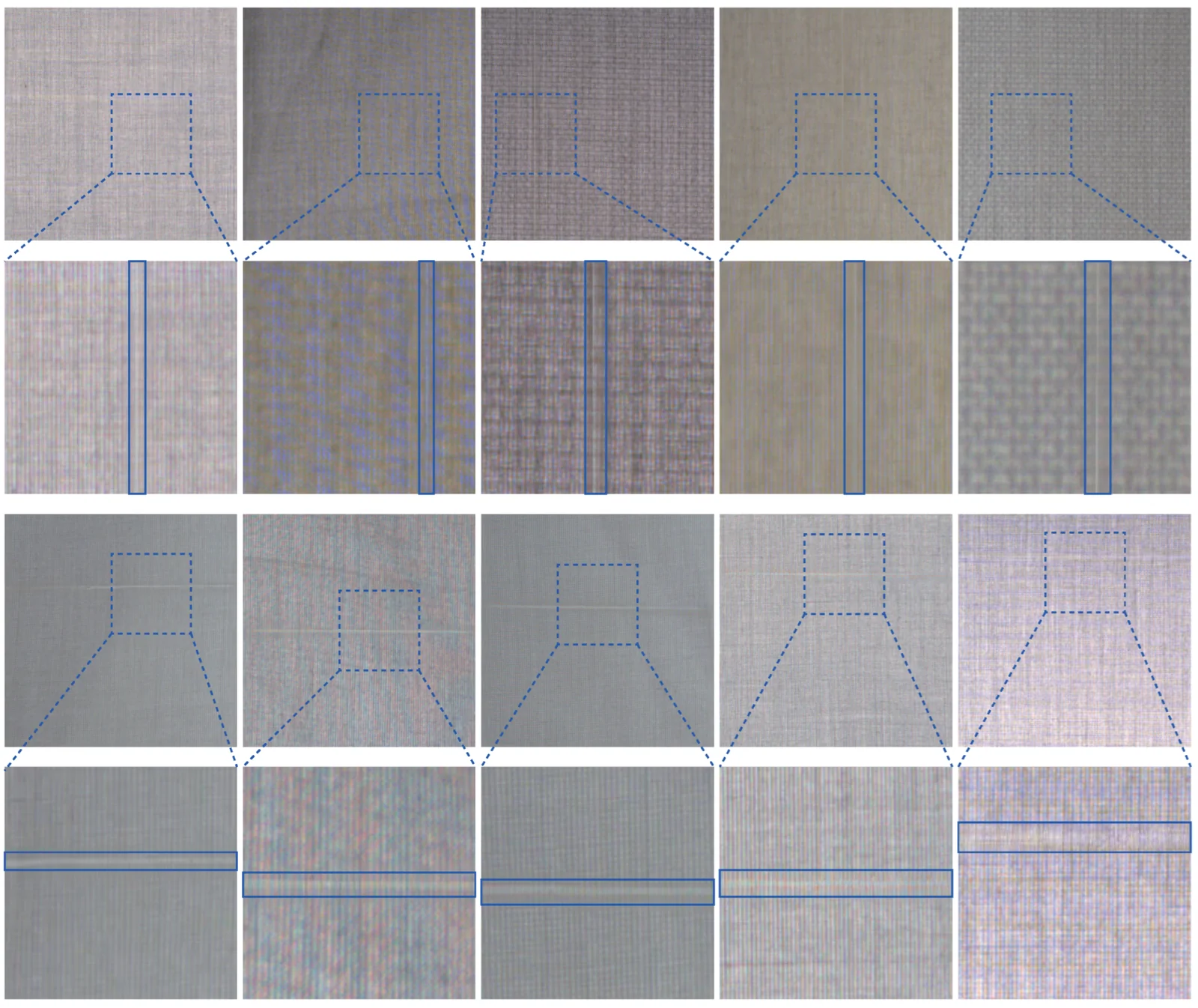
Representative SLAID-generated BrokenEnd and ThinPlace samples. The generated defects show morphological diversity, random spatial placement, and position variation across different target fabric backgrounds, thereby expanding the scale and visual diversity of the original dataset.

Representative generated results are shown in Figure 6. The synthetic BrokenEnd and ThinPlace defects exhibit variation in morphology, spatial location, and their combinations with different fabric backgrounds. These variations increase the number of visible defect appearance types and background-defect combinations during detector training. Consequently, the generated samples effectively expand the scale and visual diversity of the original limited defect dataset while preserving the typical elongated morphology of the target categories.

**Figure 7:**
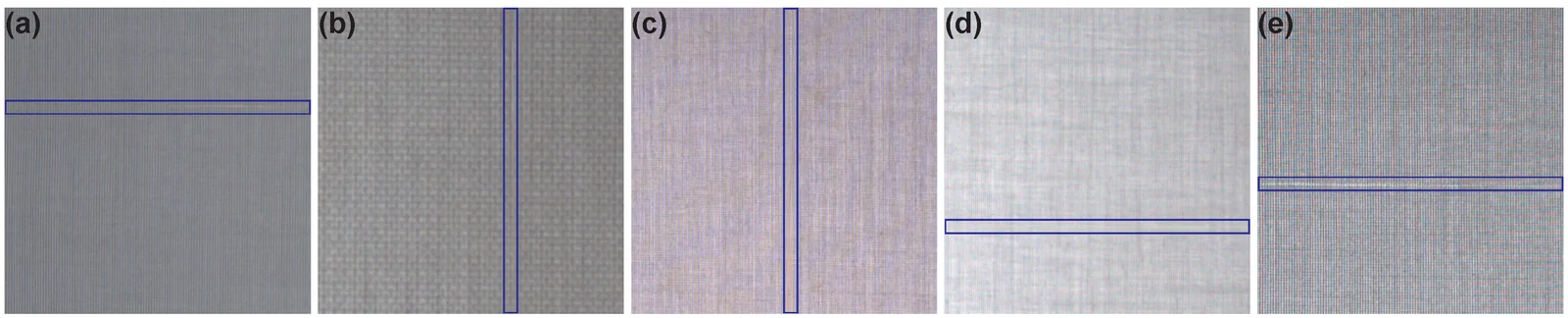
Representative failure cases of SLAID. (**a**) Incomplete generation of elongated defect structures associated with SSRM; (**b**,**c**) locally blurred defect boundaries associated with AHFG; and (**d**,**e**) imperfect defect–background blending associated with TLA.

Despite the overall generation quality, several representative failure cases remain, as shown in Figure 7. In Figure 7a, only part of the elongated defect structure is successfully synthesized, reflecting a remaining limitation in the long-range structural preservation targeted by SSRM. Figure 7b,c show locally blurred defect boundaries, indicating that the high-frequency guidance provided by AHFG may become insufficient for weak-contrast defect regions. Figure 7d,e exhibit imperfect defect–background blending, suggesting that TLA cannot completely suppress local texture inconsistency under challenging background conditions. These cases reveal the remaining limitations of SLAID in preserving complete structures, fine boundary details, and local background compatibility.

Because the preceding analysis is based mainly on visual inspection and geometric distribution comparisons, MMD^2^, LPIPS, and SSIM were further employed to quantitatively evaluate defect-distribution consistency, local perceptual similarity, and target–background preservation, respectively.

### 4.5. Ablation Study

**Figure 8:**
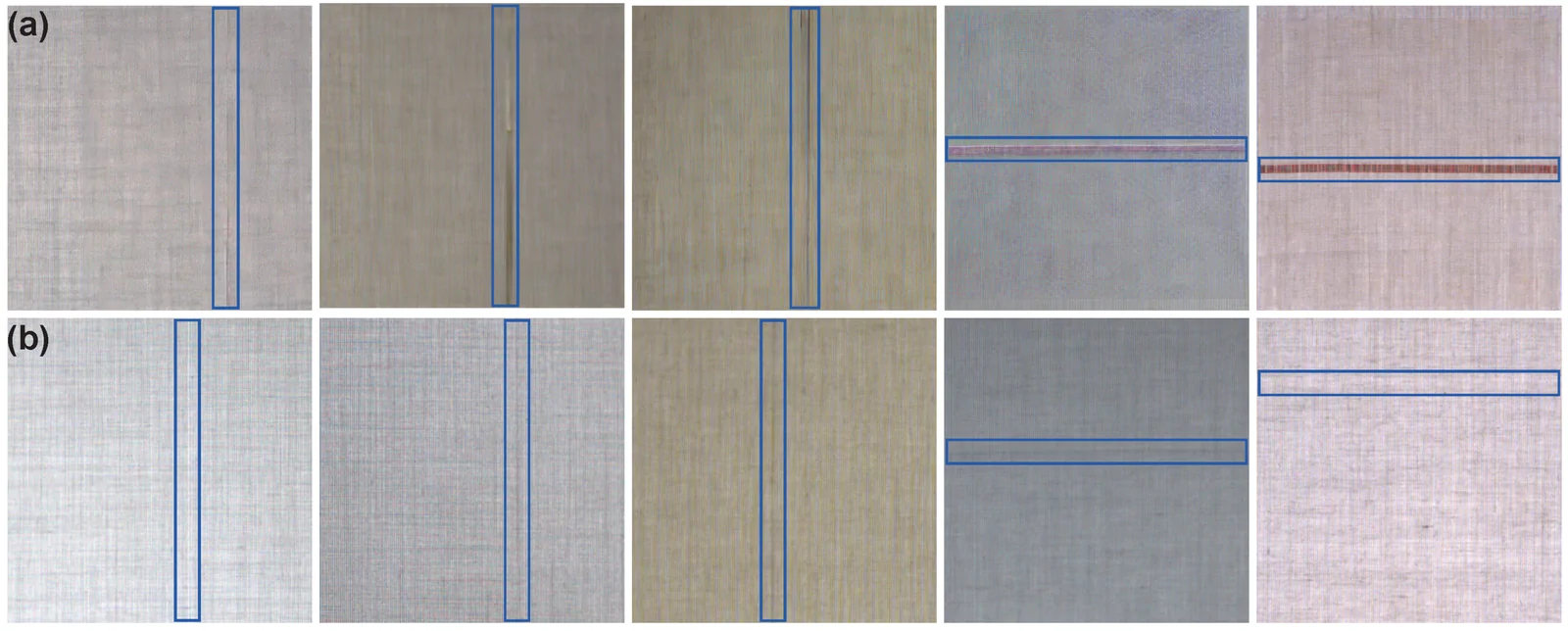
Visual comparison between the direct reference-guided baseline and real elongated fabric defects. (**a**) Baseline-generated samples. (**b**) Real defect samples. The baseline can synthesize defect-like appearances, but it tends to produce shortened or fragmented structures, blurred or abrupt weak boundaries, and local background disturbances compared with real elongated defects.

This section reports an ablation analysis of the proposed SLAID framework. To illustrate the limitations of the direct reference-guided baseline, Figure 8 compares its generated results with real slender fabric defects. The baseline can synthesize defect-like appearances on the target fabric to some extent, but clear differences remain between the generated results and real defects. In several samples, the synthesized defects show shortening, compression, or local discontinuities along the main direction, which weakens the long-range continuity required for elongated fabric defects. In addition, their boundaries are sometimes blurred or abrupt, suggesting that subtle high-frequency defect cues are not sufficiently recovered. The background texture around the edited region may also be disturbed, causing color inconsistency or texture drift.

These observations indicate that directly applying a reference-guided editing framework is insufficient for elongated fabric defect synthesis. This limitation is mainly caused by three factors: fixed-scale reference modeling cannot reliably preserve the morphology of large-span defects; coarse spatial guidance is unable to capture subtle boundary and texture variations; and the denoising process lacks explicit constraints on non-edited fabric regions, resulting in unstable background textures. Therefore, the ablation study evaluates the contributions of SSRM, AHFG, and TLA. SSRM is used to preserve long-range defect morphology, AHFG enhances weak high-frequency responses and boundary details, and TLA stabilizes non-edited target regions during DDIM sampling. The following experiments examine whether these modules can alleviate the typical failure modes observed in the direct baseline.

**Figure 9:**
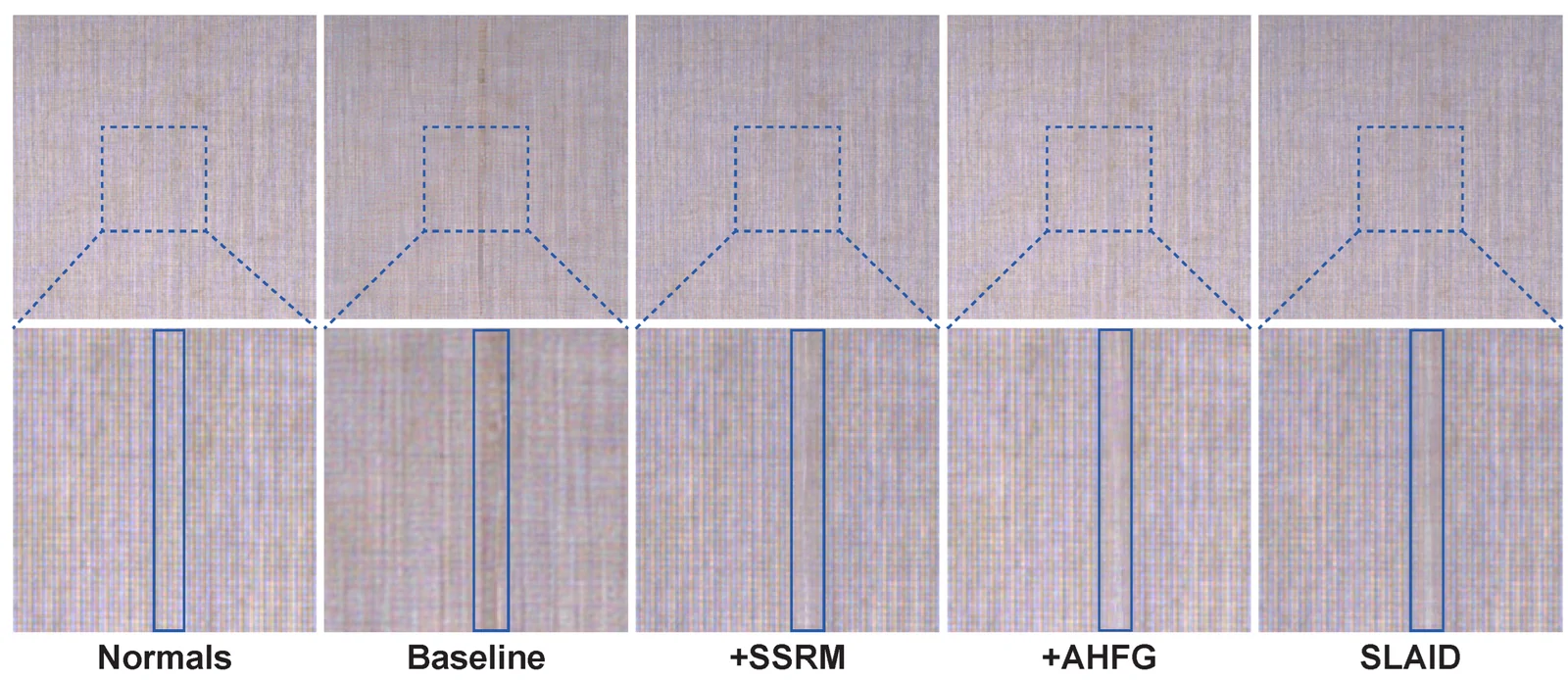
Qualitative ablation comparison of SLAID components. Compared with the reference-guided baseline, adding SSRM improves the structural continuity of elongated defects, further incorporating AHFG enhances weak local defect responses and boundary details, and the full SLAID framework with TLA produces more continuous defects with better background consistency.

As shown in Figure 9, defects generated directly by the baseline often suffer from structural fragmentation, weak local responses, and unstable background textures. Introducing SSRM improves the structural continuity of elongated defects. Further incorporating AHFG strengthens weak local defect responses, boundary details, and fine texture variations. In the complete SLAID framework, TLA improves background consistency by stabilizing non-edited target regions during DDIM sampling.

The quantitative results in Table 5 are generally consistent with the above visual observations. The direct reference-guided baseline obtains an MMD^2^ of 0.2664, an SSIM of 0.5728, and a mAP@0.5 of 0.631, indicating that direct reference-guided editing alone is not sufficient for generating elongated fabric defects. The baseline requires an average of 15.848 s to generate one image under 50-step DDIM sampling.

To further investigate the influence of the activation thresholds in SSRM, we conducted a sensitivity analysis on the span-ratio threshold ρ and aspect-ratio threshold η. Specifically, ρ was varied from 0.85 to 0.98 while fixing η=12.0, and η was varied from 8 to 16 while fixing ρ=0.93. All other experimental settings were kept unchanged. The corresponding results are reported in Table 6. The results show that the performance is relatively stable around ρ=0.93 and η=12.0, while excessively low or high thresholds may lead to inappropriate activation of multi-window reference modeling. Therefore, ρ=0.93 and η=12.0 were adopted as the default activation thresholds for SSRM.

After SSRM is incorporated, MMD^2^ decreases from 0.2664 to 0.1432, LPIPS decreases from 0.3540 to 0.1949, SSIM increases from 0.5728 to 0.9437, and mAP@0.5 improves from 0.631 to 0.768. These results show that shape-sensitive sliding-window reference modeling helps preserve the main orientation, spatial extent, and long-range continuity of elongated defects. However, SSRM introduces the largest computational overhead, increasing the runtime from 15.848 s to 22.354 s per image (+6.506 s), mainly due to the additional window-specific noise predictions.

When AHFG is further introduced on top of SSRM, MMD^2^ decreases to 0.0786, LPIPS further decreases to 0.1256, SSIM increases to 0.9524, and mAP@0.5 rises to 0.807. This improvement indicates that adaptive high-frequency guidance enhances weak defect boundaries, local texture variations, and fine-line responses, thereby improving both generation quality and downstream detection performance. AHFG adds only 0.024 s per image, indicating minimal computational overhead.

The complete SLAID framework achieves the best overall performance in the ablation experiments. With SSRM, AHFG, and TLA integrated together, MMD^2^ further decreases to 0.0435, LPIPS decreases to 0.0739, SSIM increases to 0.9754, and mAP@0.5 reaches 0.817. The SSIM improvement after adding TLA shows that target-domain latent anchoring can effectively stabilize non-edited texture regions during DDIM sampling. TLA adds only 0.106 s per image, resulting in a total runtime of 22.484 s. Overall, the three modules play complementary roles in long-span structure preservation, local detail enhancement, and background consistency constraints.

### 4.6. Comparison in Anomaly Generation

SLAID was first assessed in terms of defect-distribution consistency, local perceptual similarity and target–background preservation. As shown in Table 7, SLAID obtains the lowest mean MMD^2^ among all compared methods, with a value of 0.0435. It also achieves the lowest MMD^2^ for each of the five defect categories, namely BrokenEnd, ThickPlace, Misdraw, Reediness, and ThinPlace. These results show that, in the ResNet-50 feature space, the samples generated by SLAID are more consistent with real elongated defects than those produced by the baseline methods.

Local perceptual similarity was further evaluated using LPIPS over the 10%-expanded defect regions. As shown in Table 7, SLAID achieves the lowest mean LPIPS of 0.0739 and consistently obtains the lowest values across all five defect categories. These results indicate that the synthesized defects achieve better perceptual consistency with their corresponding real references within the local defect-context regions.

Target–background preservation was evaluated by calculating the full-image RGB SSIM between each generated image and its corresponding target image. As shown in Table 7, SLAID achieves the highest mean SSIM among the compared methods, reaching 0.9754. This result indicates that most non-edited fabric structures are retained during reference-guided defect insertion. Since full-image SSIM is mainly dominated by the background region, it is used primarily as an indicator of background preservation and is interpreted together with MMD^2^, LPIPS and qualitative results.

**Figure 10:**
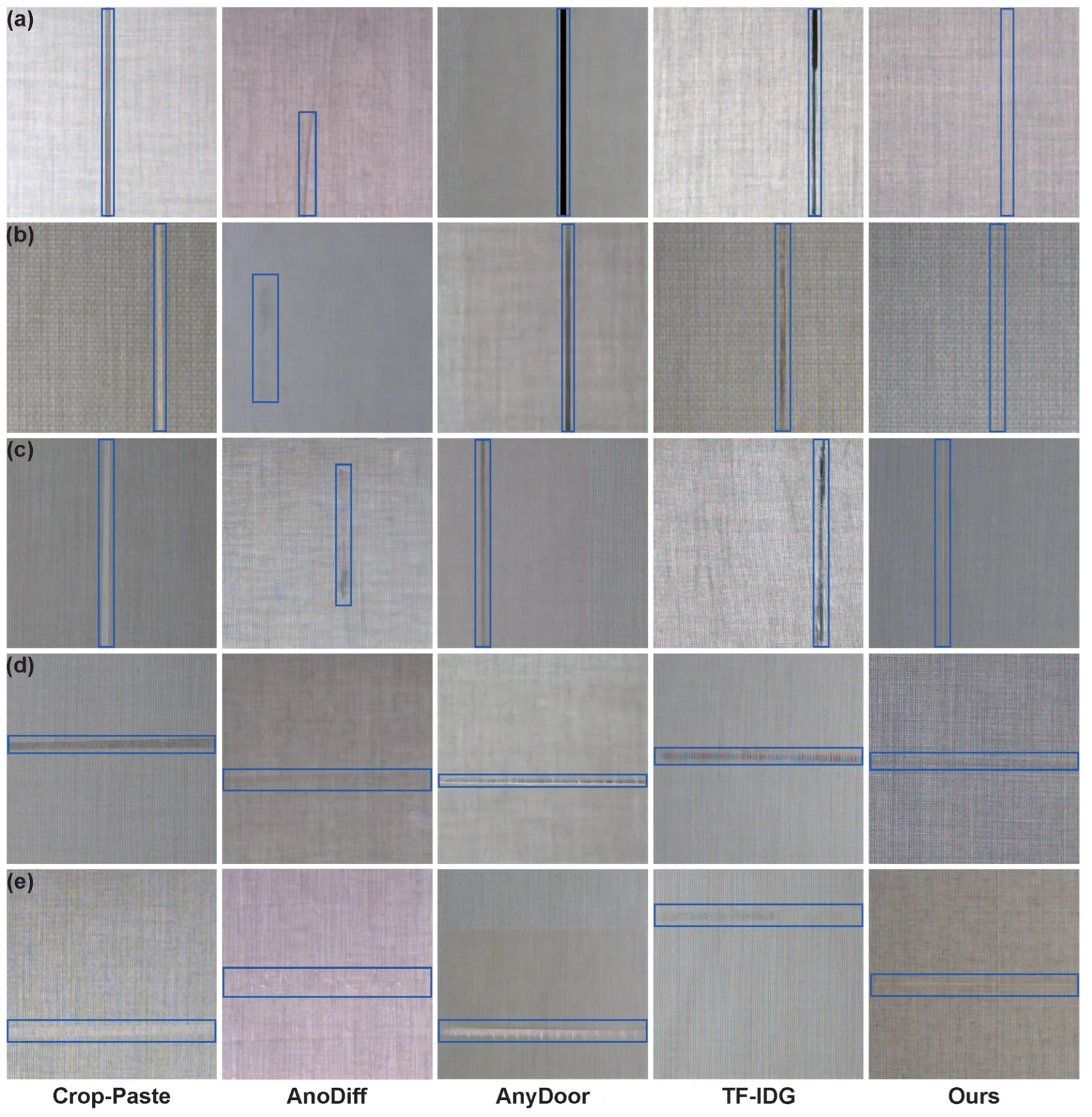
Qualitative comparison of anomaly generation methods on the ZD001 fabric defect dataset. Columns from left to right show Crop-Paste, AnoDiff, AnyDoor, TF-IDG, and the proposed SLAID. Rows (**a**–**e**) correspond to BrokenEnd, Misdraw, Reediness, ThickPlace, and ThinPlace, respectively.

The qualitative results in Figure 10 are generally in line with the quantitative comparisons. Crop-Paste can retain the appearance of the source defect, but the pasted region often shows abrupt boundaries, which limits its integration with the target fabric texture. AnoDiff and TF-IDG can generate certain defect styles; however, when dealing with long and narrow defects, their results still suffer from blurring, structural fragmentation, or unstable background textures. AnyDoor, as a strong reference-guided editing baseline, can transfer defect appearances, but direct reference transfer tends to compress some elongated structures and weaken local high-frequency details.

By contrast, the defects generated by SLAID show more continuous morphology, clearer boundaries, and better compatibility with periodic fabric backgrounds. Across the five selected categories, the generated defects generally maintain the dominant orientation and spatial extent of the reference defects. These results are consistent with the design objectives of the three modules: SSRM preserves long-range reference morphology, AHFG enhances faint high-frequency cues, and TLA constrains non-edited regions in the target latent space. Overall, SLAID improves the synthesis quality of elongated fabric defects while preserving the stability of the target background.

### 4.7. Cross-Dataset Evaluation

To evaluate whether SLAID can operate beyond the textile domain, we conducted a cross-dataset experiment on MHPSD, a steel surface defect dataset containing four categories: Bs, Foe, In, and Ss. These categories exhibit morphologies that differ substantially from the elongated fabric defects in ZD001. For each category, 200 synthetic images were generated, yielding 800 images in total. MHPSD was not used for model training, fine-tuning, or parameter optimization. MMD^2^ and LPIPS were used to evaluate defect-level distribution consistency and local perceptual similarity, respectively, while full-image RGB SSIM measured target–background preservation.

As reported in Table 8, SLAID achieves the lowest mean MMD^2^ of 0.0726 and the lowest mean LPIPS of 0.0842 among all compared methods. Crop-Paste, AnoDiff, AnyDoor, and TF-IDG obtain mean MMD^2^ values of 0.0777, 0.0996, 0.2259, and 0.1411, respectively, and mean LPIPS values of 0.0934, 0.2534, 0.3126, and 0.1780. At the category level, SLAID produces lower MMD^2^ values for In and Ss, whereas Crop-Paste performs better for Bs and Foe. Nevertheless, SLAID achieves the best overall MMD^2^ mean. Its lower LPIPS mean also indicates better local perceptual consistency with the corresponding real defect references.

For target–background preservation, SLAID obtains a mean SSIM of 0.9705, which is close to Crop-Paste at 0.9714 and higher than TF-IDG, AnoDiff, and AnyDoor, whose mean SSIM values are 0.9379, 0.6689, and 0.6203, respectively. Since full-image SSIM is dominated by unchanged background pixels, the small difference from Crop-Paste should be interpreted jointly with MMD^2^ and LPIPS. These results indicate that SLAID preserves the steel background while producing defect appearances that are more consistent with real samples.

**Figure 11:**
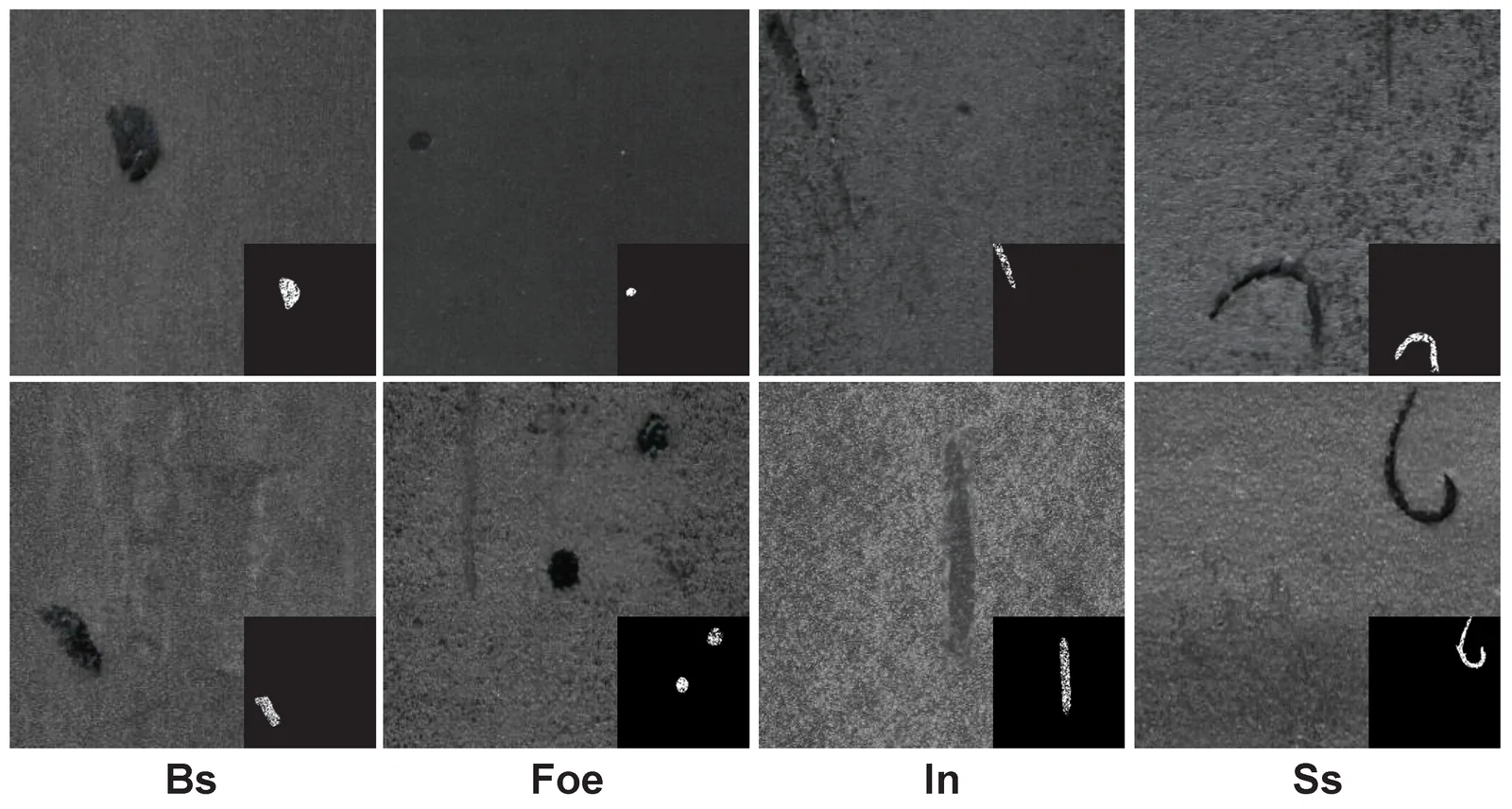
Qualitative cross-dataset generation results of SLAID on the MHPSD steel surface defect dataset. Columns correspond to the Bs, Foe, In, and Ss defect categories. Two representative generated samples are shown for each category, and the lower-right inset in each image shows the corresponding defect mask.

Qualitative examples in Figure 11 further show that SLAID can synthesize visually distinct Bs, Foe, In, and Ss defects on steel surfaces. The generated samples retain category-dependent structures, ranging from compact local anomalies to narrow and curved defects, while the surrounding surface texture remains largely unchanged. This behavior is consistent with the complementary roles of SSRM, AHFG, and TLA: SSRM maintains reference structure, AHFG strengthens weak local cues, and TLA anchors non-edited target latents.

Overall, the cross-dataset results show that the proposed inference-stage constraints remain effective when the target domain changes from fabric to steel, without dataset-specific optimization.

### 4.8. Defect Detection Results

To examine whether the generated samples are useful for practical detection rather than only visual composition, they are further used in downstream defect detection tasks. Specifically, YOLOv8 detectors are trained under two settings. The first setting uses only the original ground-truth training set, while the second adds SLAID-generated samples to the ground-truth training set for data augmentation. The two settings adopt the same network architecture, preprocessing procedure, input resolution, data augmentation strategy, and training schedule, and are evaluated on the same reserved real test set. The real training, validation, and test subsets were fixed throughout all experiments, and synthetic images were used only for training. Each detector configuration was trained five times using different YOLOv8 training seeds. For each run, the real-only and SLAID-augmented settings used the same seed, forming five seed-matched pairs, while the corresponding training datasets were kept unchanged across repeated runs. The results are reported as mean ± standard deviation over the five runs.

**Figure 12:**
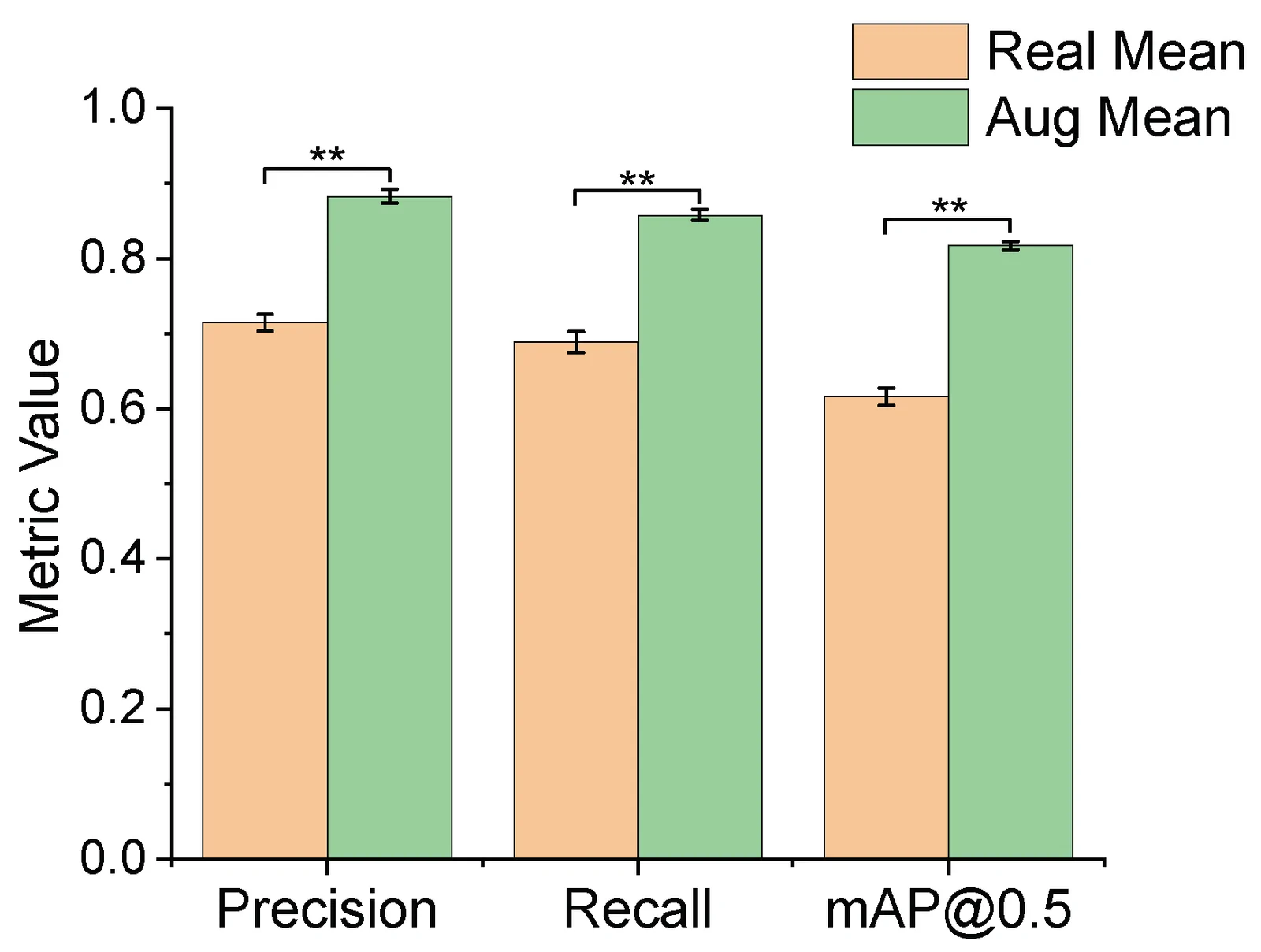
Statistical comparison of downstream detection performance between the real-only and SLAID-augmented training settings over five seed-matched runs. Bars represent the mean values, and error bars indicate standard deviations. Statistical significance was evaluated using a two-sided paired *t*-test, with ** indicating p<0.01.

As shown in Table 9, adding SLAID-generated samples improves Precision, Recall, and AP@0.5 for all five defect categories. The mean Precision increases from 0.715 ± 0.011 to 0.883 ± 0.009, the mean Recall from 0.689 ± 0.014 to 0.858 ± 0.007, and the mAP@0.5 from 0.616 ± 0.012 to 0.817 ± 0.006. The relatively small standard deviations indicate that these improvements are consistently observed across different detector-training seeds. To further assess the statistical significance of the observed improvements, a two-sided paired *t*-test was performed using the five seed-matched pairs. As shown in Figure 12, statistically significant differences were observed for Precision, Recall, and mAP@0.5 (p<0.01).

Because the increase in mAP@0.5 from 0.616 to 0.817 is relatively large, we further investigated the influence of synthetic-data quantity. For this controlled analysis, we additionally generated a larger pool of 300 synthetic images per defect category, from which subsets of 100 and 200 images were selected. The 200-image setting corresponds to the augmentation protocol used in the main experiments. As reported in Table 10, using 0, 100, 200, and 300 synthetic images per defect category yields mAP@0.5 values of 0.616 ± 0.012, 0.742 ± 0.009, 0.817 ± 0.006, and 0.826 ± 0.007, respectively. The results show a clear improvement as synthetic samples are introduced, followed by diminishing returns beyond 200 samples per category. In particular, increasing the number of synthetic images from 200 to 300 improves mAP@0.5 only marginally from 0.817 to 0.826, while Precision increase from 0.883 to 0.887 and Recall from 0.858 to 0.869.

The relatively large improvement obtained with 200 synthetic images per category should be interpreted in the context of the limited real training set. Only 100 real training images are available for each category, whereas adding 200 synthetic images increases the training set to 300 images per category. Moreover, as shown in Figure 5 and Figure 6, the generated samples preserve the characteristic elongated defect morphology while introducing variations in defect appearance, spatial position, and target fabric background. This increases the diversity of defect–background combinations available during detector training and helps alleviate the limited coverage of the original real training set.

**Figure 13:**
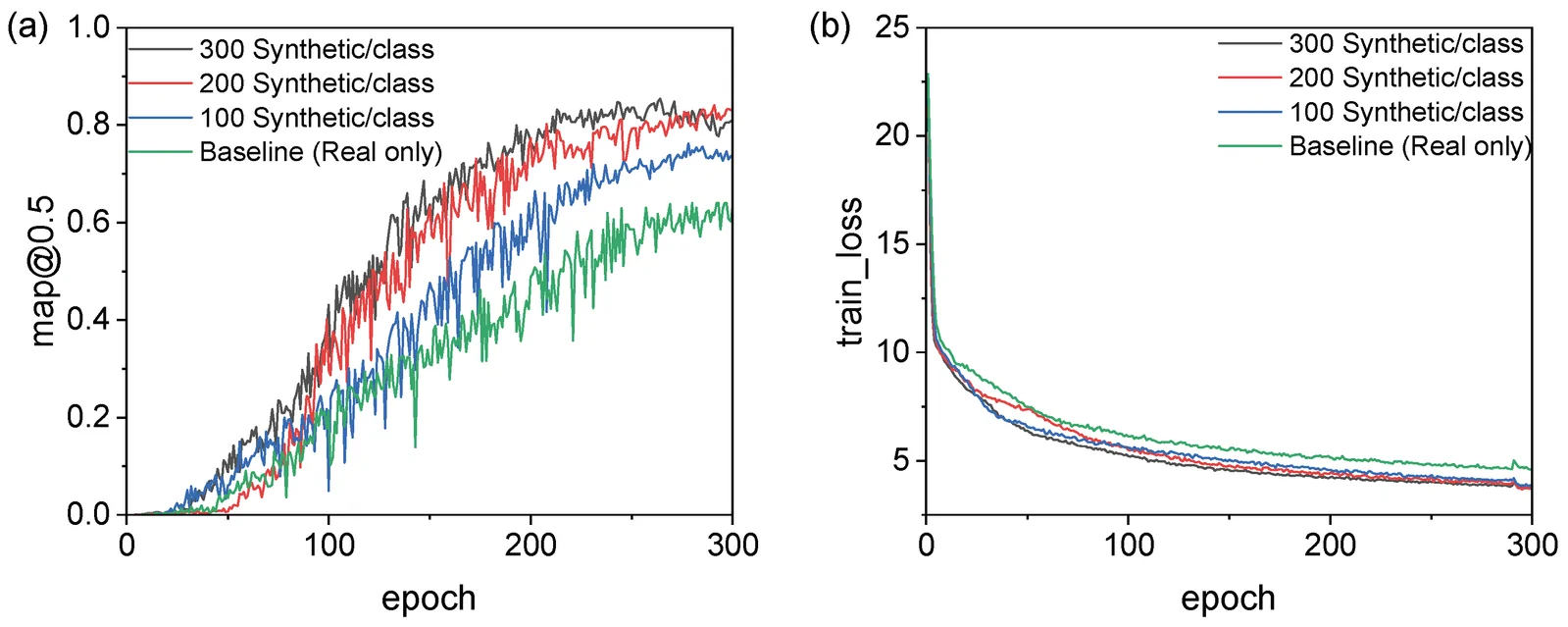
Training convergence of YOLOv8 under different synthetic-data augmentation settings. (**a**) Validation mAP@0.5 curves and (**b**) training-loss curves over 300 epochs for the real-only baseline and the settings augmented with 100, 200, and 300 SLAID-generated images per defect category.

Figure 13 further shows the corresponding convergence behavior. Compared with the real-only baseline, the augmented settings achieve higher validation mAP@0.5 during the later stages of training. The curves for 200 and 300 synthetic images per category approach similar performance levels after convergence, which is consistent with the saturation trend observed in Table 10. Meanwhile, the training-loss curves decrease smoothly without evident optimization instability. These observations further support that the improvement is associated with the additional synthetic training information rather than an isolated final-epoch fluctuation.

The improvements are especially evident for elongated fabric defects. These defects usually occupy only a small proportion of pixels, show low contrast, and extend over a long spatial range, making them susceptible to false negatives. After data augmentation, Recall improves across all categories, indicating fewer missed detections for elongated defects. Meanwhile, the increase in Precision suggests that the augmented detector not only improves detection capability, but also distinguishes real defect regions more accurately.

**Figure 14:**
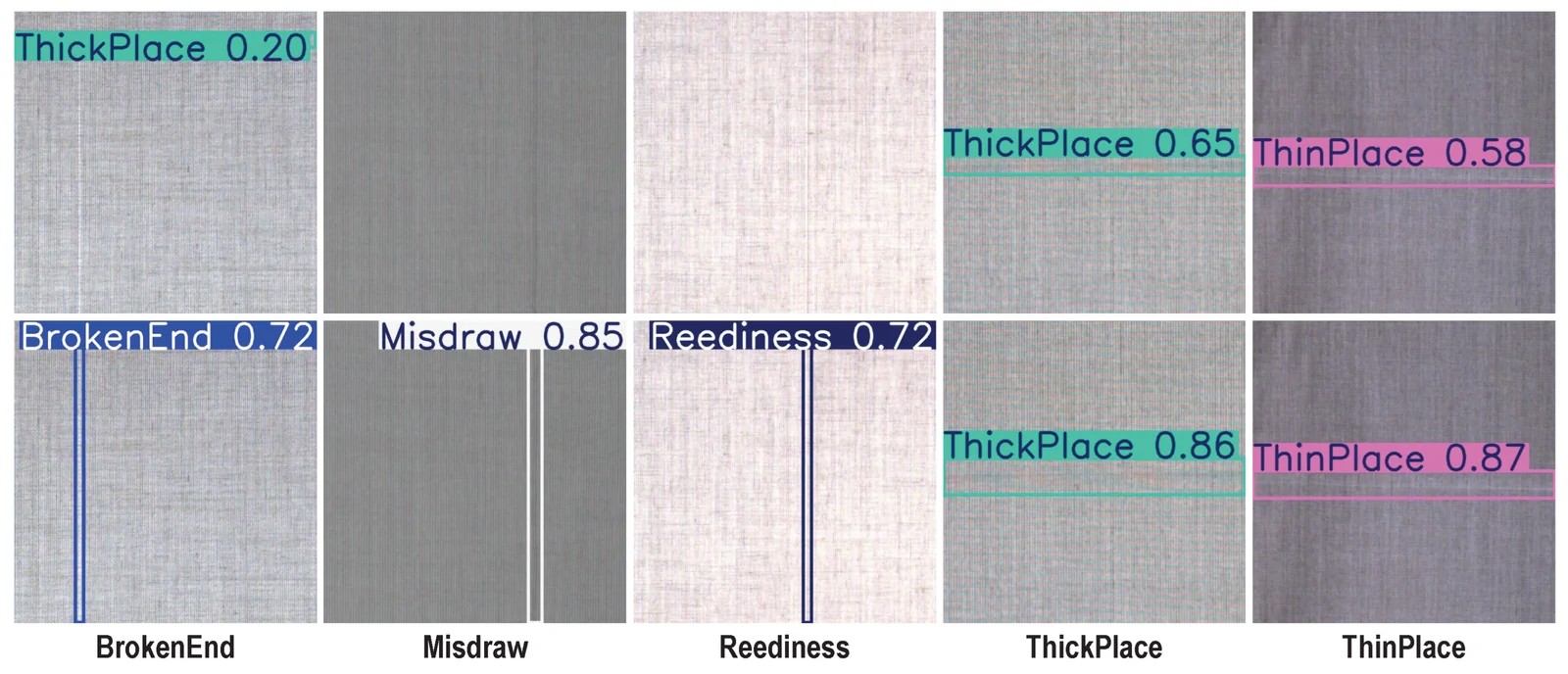
Qualitative comparison of downstream defect detection results. The detector trained with only the real dataset produces missed detections or low-confidence predictions for several elongated defects, whereas the detector trained with SLAID-augmented data achieves more complete localization and higher-confidence predictions.

The qualitative detection results in Figure 14 are consistent with the quantitative results. The detector trained only on real data produces unstable predictions, including weak responses, low confidence, missed detections, and a small number of false positives or false negatives. In contrast, the detector trained with SLAID-augmented data achieves more complete defect localization and higher-confidence predictions, while reducing visually obvious erroneous responses. Together with the repeated-run results in Table 9, the statistical analysis in Figure 12, the synthetic-data quantity study in Table 10, and the convergence analysis in Figure 13, these results demonstrate that SLAID-generated samples provide stable and effective supplementary training data for downstream fabric defect detection under limited real-data conditions.

## 5. Conclusions

We presented SLAID, a fine-tuning-free reference-guided diffusion framework for elongated fabric defect generation. SLAID integrates SSRM, AHFG, and TLA into a frozen diffusion editing backbone. These modules address three key challenges in elongated fabric defect synthesis: preserving long-range morphology, enhancing weak high-frequency details, and maintaining background consistency in non-edited fabric regions.

Experiments on five elongated defect categories from the ZD001 fabric dataset showed that SLAID achieved better defect-distribution consistency and background preservation than the compared anomaly synthesis and reference-guided generation baselines, with a mean MMD^2^ of 0.0435, a mean LPIPS of 0.0739, and a mean SSIM of 0.9754. The ablation results showed that AHFG, SSRM, and TLA contributed to local detail enhancement, long-span structure preservation, and target–background stabilization. In downstream detection, adding SLAID-generated samples increased YOLOv8 mAP@0.5 from 0.616 to 0.817 under limited real-defect training data. Evaluation on the MHPSD steel-surface defect dataset provided preliminary evidence of the applicability of the proposed inference-stage constraints to the evaluated steel-surface defect categories without dataset-specific optimization.

Several limitations remain. Incomplete long-range synthesis, blurred weak boundaries, and local defect–background inconsistencies may occur under challenging conditions, and SLAID relies on reference defect masks and target masks for structure-aware generation. Evaluation is restricted to selected categories from ZD001 and MHPSD and is insufficient to establish broad generalization across industrial materials, defect types, or acquisition conditions. The lack of machine- and production-batch-level identifiers in ZD001 prevents verification of machine- or batch-disjoint splits, leaving performance on unseen machines and batches unassessed. The absence of FID/KID and expert assessment limits the completeness of the synthesis-quality evaluation. Furthermore, SDXL-, ControlNet-SDXL-, and other newer diffusion-editing frameworks were not included; comparative conclusions are therefore restricted to the evaluated methods and configurations.

Future work will explore weaker mask supervision and broader evaluation across fabrics, machines, and production batches using datasets with appropriate acquisition metadata. Additional synthesis-quality assessment using FID/KID and expert evaluation, together with comparisons against newer diffusion-based frameworks under standardized conditioning and adaptation protocols, would further strengthen the evidence for the method’s applicability.

## Figures and Tables

**Table 1:** Training sample size before and after synthetic expansion for each elongated defect category.

Category	Sample Size (Before Expansion)	Sample Size (After Expansion)	Image Size
BrokenEnd	100	300	512×512
ThickPlace	100	300	512×512
Misdraw	100	300	512×512
Reediness	100	300	512×512
ThinPlace	100	300	512×512

**Table 2:** Sample configuration used for cross-dataset evaluation on the MHPSD steel-surface defect dataset.

Category	Sample Size (Before Expansion)	Sample Size (After Expansion)	Image Size
Bs	120	320	256×256
Foe	120	320	256×256
In	120	320	256×256
Ss	120	320	256×256

**Table 3:** Experimental Environment Configuration for Generative Models.

Environment Type	Configuration Item	Specification
Hardware	GPU Model	NVIDIA GeForce RTX 2080 Ti
	GPU Quantity	1
	CPU Model	AMD EPYC 7302 16-Core Processor
Software	Operating System	Ubuntu 20.04.5 LTS
	CUDA Version	11.8
	Deep Learning Framework	PyTorch 2.0.0

**Table 4:** Hyperparameter settings of the YOLOv8 detector used for downstream defect detection.

Configuration	Value	Note
Device	Train: 1; Val: 1	Number of devices
Resolution	640×640	Input image size
Epochs	300	Training epochs
Batch size	Train: 16	Batch size
Optimizer	SGD	Optimizer
Initial learning rate	$1\times 10^{-2}$	Initial rate
Weight decay	$1\times 10^{-2}$	Decay coefficient
Random seeds	0, 1, 11, 42, 66	Seed-matched repeated runs

**Table 5:** Ablation study of the proposed SLAID components on the ZD001 fabric defect dataset. MMD^2^, LPIPS, SSIM, and mAP@0.5 measure defect-distribution consistency, local perceptual similarity, background preservation, and downstream detection performance, respectively. Runtime is measured on an RTX 2080 Ti with 50 DDIM steps, and ΔRuntime denotes the additional cost of each component. Best results are in bold.

SSRM	AHFG	TLA	MMD^2^ ↓	LPIPS ↓	SSIM ↑	mAP@0.5 ↑	Runtime (s)	ΔRuntime (s)
–	–	–	0.2664	0.3540	0.5728	0.631 ± 0.009	15.848	–
✓	–	–	0.1432	0.1949	0.9437	0.768 ± 0.008	22.354	+6.506
✓	✓	–	0.0786	0.1256	0.9524	0.807 ± 0.006	22.378	+0.024
✓	✓	✓	**0.0435**	**0.0739**	**0.9754**	**0.817 ± 0.006**	22.484	+0.106

↓ indicates that lower values are better; ↑ indicates that higher values are better.

**Table 6:** Sensitivity analysis of the SSRM activation thresholds ρ and η.

Parameter	Threshold	MMD^2^ ↓	LPIPS ↓	SSIM ↑
*ρ*	0.85	0.0578	0.0864	0.9687
	0.90	0.0486	0.0781	0.9728
	**0.93**	**0.0435**	**0.0739**	**0.9754**
	0.95	0.0472	0.0770	0.9738
	0.98	0.0614	0.0916	0.9669
*η*	8	0.0587	0.0879	0.9684
	10	0.0489	0.0788	0.9726
	**12**	**0.0435**	**0.0739**	**0.9754**
	14	0.0468	0.0765	0.9741
	16	0.0602	0.0898	0.9678

Bold thresholds indicate the selected parameter settings, and bold metric values indicate the best results within each parameter group. ↓ indicates that lower values are better; ↑ indicates that higher values are better.

**Table 7:** Comparison of different methods in terms of defect-level generation quality, local perceptual similarity and target–background preservation on the ZD001 dataset.

Model	Metrics	Defect Categories					Mean
		BrokenEnd	Misdraw	Reediness	ThickPlace	ThinPlace	
Crop-Paste	MMD^2^ ↓	0.0839	0.1492	0.1111	0.0798	0.1119	0.1072
	LPIPS ↓	0.0937	0.1084	0.1012	0.0896	0.1135	0.1013
	SSIM ↑	0.9751	0.9679	0.9764	0.9758	0.9657	0.9722
AnoDiff	MMD^2^ ↓	0.0525	0.0823	0.0610	0.1303	0.0517	0.0756
	LPIPS ↓	0.2758	0.2907	0.2826	0.2674	0.2543	0.2742
	SSIM ↑	0.5898	0.5858	0.5876	0.5946	0.5977	0.5911
AnyDoor	MMD^2^ ↓	0.2786	0.2673	0.2679	0.2818	0.2366	0.2664
	LPIPS ↓	0.3406	0.3678	0.3515	0.3784	0.3317	0.3540
	SSIM ↑	0.5846	0.5539	0.5837	0.5814	0.5604	0.5728
TF-IDG	MMD^2^ ↓	0.2604	0.1863	0.2647	0.2832	0.1473	0.2284
	LPIPS ↓	0.1887	0.2046	0.1973	0.2125	0.1714	0.1949
	SSIM ↑	0.9469	0.9377	0.9404	0.9418	0.9377	0.9409
**SLAID**	MMD^2^ ↓	**0.0329**	**0.0724**	**0.0239**	**0.0444**	**0.0440**	**0.0435**
	LPIPS ↓	**0.0708**	**0.0827**	**0.0594**	**0.0756**	**0.0809**	**0.0739**
	SSIM ↑	**0.9763**	**0.9693**	**0.9826**	**0.9765**	**0.9721**	**0.9754**

Bold values indicate the best results for each metric within each defect category and for the mean across categories. ↓ indicates that lower values are better; ↑ indicates that higher values are better.

**Table 8:** Comparison of defect-level generation quality, local perceptual similarity, and target–background preservation among different methods on the MHPSD dataset.

Model	Metrics	Defect Categories				Mean
		Bs	Foe	In	Ss	
Crop-Paste	MMD^2^ ↓	**0.0951**	**0.0817**	0.0612	0.0726	0.0777
	LPIPS ↓	**0.1018**	0.0965	0.0837	0.0914	0.0934
	SSIM ↑	**0.9724**	**0.9741**	0.9687	0.9705	**0.9714**
AnoDiff	MMD^2^ ↓	0.1186	0.1035	0.0814	0.0947	0.0996
	LPIPS ↓	0.2683	0.2576	0.2394	0.2481	0.2534
	SSIM ↑	0.6542	0.6675	0.6813	0.6726	0.6689
AnyDoor	MMD^2^ ↓	0.2487	0.2315	0.2046	0.2189	0.2259
	LPIPS ↓	0.3264	0.3178	0.2967	0.3095	0.3126
	SSIM ↑	0.6037	0.6172	0.6348	0.6254	0.6203
TF-IDG	MMD^2^ ↓	0.1652	0.1458	0.1197	0.1336	0.1411
	LPIPS ↓	0.1915	0.1812	0.1638	0.1754	0.1780
	SSIM ↑	0.9296	0.9368	0.9451	0.9402	0.9379
**SLAID**	MMD^2^ ↓	0.1098	0.0941	**0.0386**	**0.0478**	**0.0726**
	LPIPS ↓	0.1037	**0.0915**	**0.0679**	**0.0738**	**0.0842**
	SSIM ↑	0.9628	0.9681	**0.9769**	**0.9743**	0.9705

Bold values indicate the best results for each metric within each defect category and for the mean across categories. ↓ indicates that lower values are better; ↑ indicates that higher values are better.

**Table 9:** Downstream fabric defect detection results on the ZD001 dataset. The detector is trained with either the original real dataset or the dataset augmented with SLAID-generated samples. Higher values indicate better detection performance.

Training Set	Metric	BrokenEnd	Misdraw	Reediness	ThickPlace	ThinPlace	Mean
Real dataset	Precision ↑	0.718	0.731	0.688	0.715	0.723	0.715 ± 0.011
	Recall ↑	0.645	0.716	0.650	0.707	0.727	0.689 ± 0.014
	AP@0.5 ↑	0.528	0.657	0.595	0.638	0.662	0.616 ± 0.012
Augmented dataset	Precision ↑	**0.882**	**0.906**	**0.858**	**0.883**	**0.886**	**0.883 ± 0.009**
	Recall ↑	**0.847**	**0.875**	**0.831**	**0.863**	**0.874**	**0.858 ± 0.007**
	AP@0.5 ↑	**0.806**	**0.850**	**0.780**	**0.816**	**0.833**	**0.817 ± 0.006**

Bold values indicate the better result between the two training sets for each metric and defect category, including the mean across categories. ↑ indicates that higher values are better.

**Table 10:** Effect of the number of synthetic images per class on downstream defect detection performance.

Synthetic Images/Class	Precision	Recall	mAP@0.5
0	0.715 ± 0.011	0.689 ± 0.014	0.616 ± 0.012
100	0.814 ± 0.011	0.775 ± 0.012	0.742 ± 0.009
200	0.883 ± 0.009	0.858 ± 0.007	0.817 ± 0.006
300	0.887 ± 0.009	0.869 ± 0.010	0.826 ± 0.007

## Data Availability

The ZD001 dataset used in this study is available on Mendeley Data at https://doi.org/10.17632/7bw2hngcvz.1. The MHPSD dataset is accessible through https://github.com/clovermini/MVIT_metal_datasets (accessed on 30 September 2026).
